# A Network of Sporogenesis-Responsive Genes Regulates the Growth, Asexual Sporogenesis, Pathogenesis and Fusaric Acid Production of *Fusarium oxysporum* f. sp. *cubense*

**DOI:** 10.3390/jof10010001

**Published:** 2023-12-19

**Authors:** Songmao Lu, Huobing Deng, Yaqi Lin, Meimei Huang, Haixia You, Yan Zhang, Weijian Zhuang, Guodong Lu, Yingzi Yun

**Affiliations:** 1 State Key Laboratory of Ecological Pest Control for Fujian and Taiwan Crops, College of Plant Protection, Fujian Agriculture and Forestry University, Fuzhou 350001, China; songmaolu@163.com (S.L.); huobingdeng@163.com (H.D.); yqyq017@163.com (Y.L.); huangmeimei06@163.com (M.H.); haixia_you@163.com (H.Y.); zhang18328169310@outlook.com (Y.Z.); weijianz1@163.com (W.Z.); 2 Fujian Institute of Tropical Crops, Zhangzhou 363001, China; 3 Fujian Provincial Key Laboratory of Plant Molecular and Cell Biology, Oil Crops Research Institute, Fujian Agriculture and Forestry University, Fuzhou 350001, China; 4 Fujian Key Laboratory for Monitoring and Integrated Management of Crop Pests, Fuzhou 350001, China

**Keywords:** asexual reproduction, *Fusarium oxysporum* f. sp. *cubense*, genes, sporogenesis regulation

## Abstract

The conidia produced by *Fusarium oxysporum* f. sp. *cubense* (Foc), the causative agent of Fusarium Wilt of Banana (FWB), play central roles in the disease cycle, as the pathogen lacks a sexual reproduction process. Until now, the molecular regulation network of asexual sporogenesis has not been clearly understood in Foc. Herein, we identified and functionally characterized thirteen (13) putative sporulation-responsive genes in Foc, namely *FocmedA(a)*, *FocmedA(b)*, *abaA-L*, *FocflbA*, *FocflbB*, *FocflbC*, *FocflbD*, *FocstuA*, *FocveA*, *FocvelB*, *wetA-L*, *FocfluG* and *Foclae1*. We demonstrated that *FocmedA(a)*, *abaA-L*, *wetA-L*, *FocflbA*, *FocflbD*, *FocstuA*, *FocveA* and *Foclae1* mediate conidiophore formation, whereas *FocmedA(a)* and *abaA-L* are important for phialide formation and conidiophore formation. The expression level of *abaA-L* was significantly decreased in the Δ*FocmedA(a)* mutant, and yeast one-hybrid and ChIP-qPCR analyses further confirmed that FocMedA(a) could bind to the promoter of *abaA-L* during micro- and macroconidiation. Moreover, the transcript abundance of the *wetA-L* gene was significantly reduced in the Δ*abaA-L* mutant, and it not only was found to function as an activator of micro- and macroconidium formation but also served as a repressor of chlamydospore production. In addition, the deletions of *FocflbB*, *FocflbC*, *FocstuA* and *Foclae1* resulted in increased chlamydosporulation, whereas *FocflbD* and *FocvelB* gene deletions reduced chlamydosporulation. Furthermore, *FocflbC*, *FocflbD*, *Foclae1* and *FocmedA(a)* were found to be important regulators for pathogenicity and fusaric acid synthesis in Foc. The present study therefore advances our understanding of the regulation pathways of the asexual development and functional interdependence of sporulation-responsive genes in Foc.

## 1. Introduction

Fusarium Wilt of Banana (FWB), caused by the ascomycete soilborne pathogen *Fusarium oxysporum* f. sp. *cubense* (Foc), is the most destructive disease of bananas worldwide [1,2]. Based on its pathogenicity to different banana reference varieties, Foc can be classified into race 1 (R1), race 2 (R2) and race 4 (R4) [3,4,5]. Foc R4 has been further separated into Foc tropical race 4 (Foc TR4) and subtropical race 4 (SR4) [6,7]. Foc TR4 infects not only R1-susceptible varieties (‘Gros Michel’, ‘Silk’ and ‘Pisang Awak’) and R2-susceptible varieties (‘Bluggoe’) but also the ‘Cavendish’ varieties that make up 40% of the global banana production [2]. Nowadays, Foc TR4 infection is common in Asia, Oceania, Africa and America [1], and its alarming expansion has gained great interest in many banana-producing communities [8].

Due to the absence of a teleomorph [9], Foc only undergoes an asexual reproduction process, forming three kinds of spores, namely microconidia, macroconidia and chlamydospores, which are important inoculum sources for the infection and spread of FWB [10]. The microconidia are ellipsoid in shape, having 0–3 septa each; the macroconidia are falcate, possessing 3–6 septa, apical papilla cells and foot-shaped basal cells; and the chlamydospores are rough-walled globose to sub-globose [11]. The microconidia are detached from the phialides of vegetative hyphae [1], and the macroconidia are produced from the phialides of conidiophores, whereas chlamydospores are generated from hyphae or from inside the fungal conidia [12]. After penetration into banana roots through the intercellular space of the epidermis and/or wounds (or cuts), Foc begins to release microconidia and chlamydospores into the host intra- and intercellular spaces, which serve as secondary inocula for the subsequent spread of the fungus to neighboring host plants [13]. Chlamydospores can survive for more than ten years in soil, even under adverse environmental conditions [14,15,16,17]. Currently, there are no effective fungicides that satisfactorily control FWB, and there is no available Foc-resistant variety of banana; agricultural control methods (such as crop rotation) that reduce the amount of the pathogen in the soil are much more effective in the field. Therefore, identification and functional analysis of the key regulators of sporogenesis will be helpful for unveiling promising targets for FWB control.

Asexual sporulation is the most common mode of reproduction in many filamentous fungi [18], such as the model fungus *Aspergillus nidulans*. The asexual reproduction processes of *A. nidulans* have been adequately investigated over the years [19,20], and the determining factors and pathways regulating its conidiation (such as the central regulatory pathway (CRP), upstream developmental activators (UDAs), Velvet regulators, negative repressors and developmental modifier genes) have been established [19,21,22,23,24,25,26,27,28,29]. Generally, in the *Aspergillus* genus, the three transcription factors *brlA*, *abaA* and *wetA* serve as CRP components that promote successful conidiophore development and spore maturation [20,28,30,31]. However, UDAs are encoded by *fluG*, *flbA*, *flbB*, *flbC*, *flbD* and *flbE* genes, whose inactivation downregulates the expression of the CRP gene *brlA* and consequently affects the fungal transition from vegetative growth to asexual reproduction [20,27,32]. Members of the Velvet family of proteins, namely VeA, VelB, VelC and VosA, are fungi-specific transcription factors that play important roles in coordinating fungal development and secondary metabolism [33,34]. Specifically, *velB* positively regulates conidiation, unlike *veA*, *velC* and *vosA* (plus the conidiation repressors *sfgA* and *nsdD*), which negatively regulate conidia production in *A. nidulans* [18]. In the dark, *laeA* (loss of *aflR* expression), a global regulator of secondary metabolism, could form a trimeric complex with *veA* and *velB* inside the nucleus to mediate fungal development and secondary metabolism [35,36,37,38,39]. The development of the modifier genes *stuA* and *medA*, which spatiotemporally regulate the expression of the CRP genes *brlA*, *abaA* and *wetA*, is required for proper differentiation of conidiophores and phialides [40,41,42,43].

Based on the regulation network for asexual reproduction in the *Aspergillus* genus, homologs of the various regulatory elements (including *stuA*, *medA* and the Velvet regulators) in *A. nidulans* have been identified in *F. oxysporum*. In *F. oxysporum* f. sp. *melonis* (Fom) isolates, deletion of *FostuA* does not affect microconidium formation but abolishes conidiophore production with increased chlamydospore formation, suggesting that *FostuA* acts as a positive and negative regulator for the production of macroconidia and chlamydospores, respectively [44]. Moreover, deletion of *Ren1*, the homolog of *medA* in Fom, perturbs the development of phialides and conidiophores but does not block chlamydospore formation [12]. In *F. oxysporum* f. sp *lycopersici* (Fol), the *velvet* gene deletion mutants Δ*veA* and Δ*velB* produce large-sized and increased amounts of microconidia; Δ*velC* also shows increased conidiation but produces smaller microconidia, whereas Δ*laeA* produces fewer microconidia with a normal morphology [45], suggesting that the Velvet components play different roles in regulating conidiation. However, the role of the Velvet complex in relation to the production of macroconidia and chlamydospores remains to be investigated. Three kinds of asexual spores are produced in *F. oxysporum*, but only one type of conidium is produced on the conidiophores of *A. nidulans*. Therefore, it is obvious that the regulators of conidiation have functional diversity between *A. nidulans* and *F. oxysporum* fungi.

To further understand the regulation mechanisms of asexual reproduction in Foc, we examined the homolog genes of 16 sporogenesis-related genes in *A. nidulans*, and 13 genes in Foc were finally identified. We found that 11 out of the 13 genes were important for asexual reproduction in Foc, and they also participated in the vegetative growth, pathogenesis and fusaric acid production of Foc. Based on our work, a putative network of sporogenesis-responsive genes was summarized, which provides in-depth information for further understanding of the reproduction regulation process of *F. oxysporum*.

## 2. Materials and Methods

### 2.1. Fungal Strain and Media

*Fusarium oxysporum* f. sp. *cubense* tropical race 4 (Foc TR4) strain 58, derived from our previous studies and kept in our lab [46,47], was used as the wild type (WT) and as the background for all targeted gene deletions in this study. All Foc TR4 strains were stored in cryopreservation tubes with 20% glycerol solution (*v*/*w*) at −80 °C. Complete medium (CM: 20 × nitrate salts 50 mL, D-glucose 10 g, peptone 2 g, casamino acid 1 g, yeast extract 1 g, 1000 × trace elements 1 mL, 1000 × vitamin solution 1 mL, agar 20 g, distilled water 1 L) and potato dextrose agar (PDA: potato 200 g, dextrose 20 g, agar 20 g, distilled water 1 L) were used for vegetative growth assays [48,49]. Potato dextrose broth (PDB: potato 200 g, dextrose 20 g, distilled water 1 L), Spezieller Nährstoffarmer agar (SNA: D-glucose 0.2 g, sucrose 0.2 g, KH_2_PO_4_ 1 g, KNO_3_ 1 g, MgSO_4_·7H_2_O 0.5 g, KCl 0.5 g, agar 20 g, distilled water 1 L) and liquid Spezieller Nährstoffarmer (SN: D-glucose 0.2 g, sucrose 0.2 g, KH_2_PO_4_ 1 g, KNO_3_ 1 g, MgSO_4_·7H_2_O 0.5 g, KCl 0.5 g, distilled water 1 L) were used for conidiation assays [48,50]. Minimal medium (MM: NaNO_3_ 6 g, KCl 0.52 g, MgSO_4_·7H_2_O 0.312 g, KH_2_PO_4_ 1.52 g, thiamine 0.01 g, 1000 × trace elements 1 mL, D-glucose 10 g, agar 20 g, distilled water 1 L) was used for cellophane membrane penetration assays. Czapek–Dox broth (sucrose 30 g, NaNO_3_ 2 g, K_2_HPO_4_ 1 g, MgSO_4_·7H_2_O 0.5 g, KCl 0.5 g, FeSO_4_·7H_2_O 0.01 g, distilled water 1 L) was used for fusaric acid production assays [51,52].

### 2.2. Gene Identification

The protein sequences for conidiation-associated proteins in *Aspergillus nidulans* were downloaded from UniProt (https://www.uniprot.org/uniprot/, accessed on 1 December 2021). The target genes in Foc were identified by utilizing the BLASTp system in the NCBI *F. oxysporum* f. sp. *cubense* tropical race 4 (FocTR4) genome database (https://www.ncbi.nlm.nih.gov/, accessed on 1 December 2021) using the amino acid sequences of the homologous proteins of *A. nidulans.*

### 2.3. Mutant Generation

The PCR constructs for gene deletion, which comprised 1000 bp of the upstream sequence of the target gene, hygromycin phosphotransferase (*hph*) gene and 1000 bp of the downstream sequence of the target gene, were generated via the double-joint PCR approach [53]. The upstream and downstream fragments of the target genes were amplified using their specific primer pairs listed in Table S1. The PCR constructs were transformed into the protoplasts of the wild-type strain, as described previously [52,54]. CM containing hygromycin B (Beijing Lablead Biotech Co. Ltd.) at a final concentration of 150 μg/mL was used for screening of the transformants. The right mutants were identified using PCR and further confirmed via Southern blot assay using a DIG high prime DNA detection kit (Roche), according to the manufacturer’s instructions.

For complementation of Δ*FocmedA(a)* strain, the entire coding sequence of the target gene, including its native promoter region, was amplified using PCR and inserted into an *Xho*I-digested pYF11 vector using the yeast gap repair approach [55]. The construct was transformed into protoplasts of the Δ*FocmedA(a)* mutant, and the transformants were screened on CM containing G418 (150 μg/mL) as a selective marker. The complementation of Δ*FocmedA(a)* strain (Δ*FocmedA(a)-C*) was used for Chromatin Immunoprecipitation-qPCR assay.

### 2.4. Vegetative Growth and Hydrophobicity Assays

For vegetative growth assays, mycelial plugs (0.6 cm in diameter) from a 5-day-old colony of the wild-type Foc strain 58 and the mutants were inoculated at the center of PDA plates and incubated at 26 °C for 3 days [46]. The colony diameters of the various cultures were then measured and recorded. To analyze the hydrophobicity of the aerial hyphae, 30 μL of 1% acid fuchsin solution (*w*/*v*) was placed on the surface of a 3-day-old colony of each of the various strains incubated on CM plates [56]. The colonies were incubated for 10 min, after which the extent of the spread of the liquid droplets was observed and photographed using a camera (Canon).

### 2.5. In Vitro and in Planta Sporulation Assay

In vitro sporulation: For microconidia assays, three mycelial plugs (0.6 cm in diameter) were chopped and inoculated into 50 mL PDB and incubated in a shaker (Shanghai Zhichu Instrument Co., Ltd.) operating at 180 rpm at 28 °C for 3 days (with 12 h light–dark cycle). The culture was then filtered, and the number of microconidia was counted using a hemocytometer (Shanghai Qiujing Biochemical Reagent Instrument Co., Ltd.) [52]. For a macroconidia assay, the mycelial plugs were cultured on SNA plates and incubated at 26 °C [11]. After 10 days, the cultures were washed with 5 mL sterile double-distilled water (ddH_2_O) and filtered. The macroconidia in the filtrate were counted under a light microscope with the help of a hemocytometer. To assay for chlamydospores, 9 mycelial plugs (0.9 cm in diameter) were randomly removed from the colony of each strain after 10 days of inoculation on SNA. The plugs were placed in 10 mL centrifuge tubes containing 5 mL of sterile ddH_2_O. Subsequently, the mycelial plugs were crushed into fine pieces with tweezers. The hyphal tissues were further homogenized to release chlamydospores from the hyphae via ultrasonication (UP-250, Niboscientz biotechnology Co., Ltd.; on 50 s, off 10 s, 10 cycles, power 40%). The counting of chlamydospores was performed using a hemocytometer. The quantity of chlamydospores produced (spores /cm^2^) was evaluated using the following formula:

$$\mathrm{Chlamydospores}\;\mathrm{yield}\;(\mathrm{spores}\;/{\mathrm{cm}}^{2})=\frac{Concentration\;of\;chlamydospores\left(spores/\;mL\right)\times 5\;mL}{Area\;of\;9\;mycelial\;plugs\;({cm}^{2})}$$

In planta sporulation: Mycelial plugs (0.6 cm in diameter) from each strain were inoculated on detached banana roots (length: 4–5 cm, width: 5–6 mm) and incubated at 26 °C for 10 days. Some banana roots were inoculated with sterile CM plugs to serve as a negative control. After 10 days of incubation, the roots were ground in 2 mL of sterile ddH_2_O using a sterile mortar and pestle. The counting of conidia was performed under a light microscope using a hemocytometer. The quantity of conidia produced (spores/g) was evaluated using the following formula:

$$\mathrm{Conidia}\;\mathrm{yield}\;(\mathrm{spores}/g)=\frac{Concentration\;of\;conidia\left(spores/\;mL\right)\times 2\;mL}{Weight\;of\;banana\;root\;(g)}$$

To observe the formation of microconidia and macroconidia by Foc on the banana petioles, mycelial blocks (0.6 cm in diameter) were inoculated on one side of the banana petioles (length: 3–4 cm, width: 5–6 mm) and incubated at 26 °C for 15 days. The epidermis of the banana petioles was peeled off with forceps and was transferred to a glass slide containing sterile water, and the glass slides were observed using a confocal microscope and photographed.

### 2.6. Microscopy

The morphologies of the various asexual structures (including the spores, phialides and conidiophores) were investigated using a laser-scanning confocal microscope (A1R, Nikon, Tokyo, Japan).

### 2.7. Quantitative Real-Time PCR (qRT-PCR)

For gene expression assays, WT tissues at different developmental stages, including vegetative mycelia (VM: mycelia incubated in liquid CM for 24 h), reproductive mycelia from the PDB culture (RM-PDB: mycelia incubated in PDB for 3 days at 180 rpm), reproductive mycelia from the SN culture (RM-SN: mycelia incubated in SN for 6 days at 180 rpm), conidia (CO) from the RM-SN culture and germinated conidia (GC) from liquid CM, were used for total RNA extraction.

To investigate the functional interdependence of the conidiation-responsive genes in Foc, the WT and mutant strains were cultured in PDB (or SN) under constant shaking at 180 rpm at 28 °C for 3 (or 6) days. Mycelia were collected via filtration through a Miracloth for RNA extraction.

Total RNA was extracted using an Eastep^®^ super total RNA extraction kit (Shanghai Promega biological products Co., Ltd.) according to the manufacturer’s instructions. From each sample, 1 μg RNA was used for cDNA synthesis via reverse transcription using a Hiscript^®^ III RT SuperMix for qPCR (+gDNA wiper) (Vazyme Biotech Co., Ltd.). The qRT-PCR was performed with a ChamQ Universal SYBR qPCR Master Mix (Vazyme Biotech Co., Ltd.) using the Eppendorf Master cycler Ep Gradient Realplex^2^ PCR system. *Focactin* was used as an internal control [51]. The relative expressions of the transcripts were computed using the 2^−ΔΔCt^ method [57]. The primers used for the qRT-PCR analysis are listed in Table S2.

### 2.8. Yeast One-Hybrid (Y1H) Assay

To elucidate the binding of FocMedA(a) to the *abaA-L* promoter, Y1H assay was performed using the Matchmaker™ One-Hybrid Library Construction & Screening Kit (Clontech Laboratories, Inc.) following the manufacturer’s instructions. The four different fragments of the *abaA-L* promoter, as shown in Figure S1, were cloned into the pAbAi vector. The vectors were transformed into a Y1HGold yeast strain, and transformants were selected on (SD)-Ura media containing 100 ng/mL AbA (Aureobasidin A). The full length of FocMedA(a) amplified from the WT cDNA was cloned into the pGADT7 vector and subsequently transformed into different Y1HGold strains harboring the different pAbAi vectors. Interactions of FocMedA(a) with the different fragments of the *abaA-L* promoter were detected on SD-Leu media containing 100 ng/mL AbA. The *AbaA-L*-p-AbAi and pGADT7 plasmids were used as negative controls. The experiment was independently repeated three times.

### 2.9. Chromatin Immunoprecipitation (ChIP)-qPCR Assay

ChIP experiments were performed following previously published protocols with minor modifications [58,59]. Firstly, fresh mycelia from Δ*FocmedA(a)-C* (Table S3) were incubated in 1% formaldehyde at 180 rpm at 28 °C for 10 min and further incubated in 125 mM glycine for 5 min. The mycelia were then collected and ground to powder in liquid nitrogen and transferred to lysis buffer containing a protease inhibitor cocktail (Roche). DNA was sheared into 200–500 bp fragments using a Bioruptor^®^ Plus sonication device (30 s on and 30 s off) (Diagenode, Catalog Number B01020001). Immunoprecipitation was conducted using the Anti-GFP antibody ab290 (Abcam) or Mouse IgG1 (Invitrogen, MA1-10406) together with protein A–agarose beads (sc-2001, Santa Cruz Biotechnology, Inc.). The beads were subsequently washed with a low-salt wash buffer, high-salt wash buffer, LiCl wash buffer and TE buffer [59]. The immunoprecipitated samples were eluted from the beads using an elution buffer. The resulting complexes were subsequently treated with 5 µL RNase at 45 °C for 1.5 h, and the complexes were reverse cross-linked and digested with proteinase K at 45 °C for 2 h, after which an equal volume of phenol/chloroform/isoamyl alcohol was added to precipitate DNA at −80 °C for 1 h. The enriched DNA was used for a quantitative PCR (qPCR) analysis using a ChamQ Universal SYBR qPCR Master Mix (Vazyme Biotech Co., Ltd.) and specific primer pairs (Table S2). Relative enrichment was calculated by normalizing the values of the immunoprecipitated samples with those of the inputs. The experiment was conducted three times independently.

### 2.10. Pathogenicity Assays in Banana Plants

Susceptible ‘Cavendish’ banana (AAA) plantlets with four to five leaves were cultivated in a greenhouse (light: 16 h; temperature: 26 °C; relative humidity: 75%) for pathogenicity assays. The banana roots were aseptically wounded using sterile 2 cm cut tips and immersed in a conidial suspension at a concentration of 5 × 10^6^ microconidia/mL and incubated overnight in a shaker operating at 90 rpm [48]. Because Δ*abaA-L* could not produce any conidium, we inoculated the banana roots with a homogenized mycelia suspension of Δ*abaA-L* instead of the conidia solution, and a similar mycelia suspension from the wild-type strain was used as a control. Subsequently, the inoculated banana plants were replanted in the greenhouse for 2 months. The inner rhizome and the inner rhizome with brown necrosis were photographed with a camera, and their areas were analyzed using ImageJ software [60]. To establish the disease severity, the percent of inner rhizome showing browning was calculated using the following formula:

$$\frac{Area\;of\;the\;inner\;rhizome\;showing\;browning}{Area\;of\;the\;total\;inner\;rhizome}\times 100\%$$

### 2.11. Cellophane Membrane Penetration Assays

The surfaces of MM medium plates were covered with sterile cellophane membranes, and 5 μL of conidial suspension (5 × 10^6^ spores/mL) from each strain was dropped at the center of the membranes, respectively, and incubated at 26 °C for 3 days. Subsequently, the cellophane membranes with the mycelial mass were removed, and the MM medium plates were further incubated for 2 days. The presence of fungal hyphae growing on the MM medium plates indicated successful penetration of the membrane [52,61].

### 2.12. Fusaric Acid Production Assays

The amount of fusaric acid (FA) produced by the fungal strains was measured using high-performance liquid chromatography (HPLC) (Agilent 1100 Series), as described previously, with minor modifications [51,52]. The fungal strains were inoculated in 50 mL liquid Czapek–Dox medium and incubated in a rotary shaker running at 120 rpm at 26 °C for 15 days [51,52]. Subsequently, mycelia were collected using filtration and dried in an oven (DHG-9053BS-Ⅲ, Shanghai CIMO Medical Instrument Manufacturing Co., Ltd.) at 65 °C for 2 days. Meanwhile, the filtrates were centrifugated (Centrifuge 5430 R, Eppendor) at 5000 rpm for 3 min. The supernatants were collected and adjusted to pH 3.0 using concentrated hydrochloric acid (HCl), and they were partitioned in ethyl acetate. Eventually, the ethyl acetate extracts were dried in a nitrogen evaporator (Digital Dry Bath, Hangzhou Ruicheng Instrument Co. Ltd.), and the residues were redissolved in 1 mL methanol and filtered through a 0.45 µm membrane. An Agilent 1100 LC system with a Diamonsil^®^-C18 (150 × 4.6 mm, 5 μm) was used for a fusaric acid analysis; gradient elution was performed using a mobile phase containing methanol and 30 mM H_3_PO_4_ (pH 7.34) for 25 min at a flow rate of 1 mL/min with a UV detector at 270 nm. The FA content of each strain is presented in mg/g dry mycelia weight [51,52]. The experiment was performed three times.

### 2.13. Statistical Analyses

Each experiment was performed triplicate and values are presented as means ± standard deviations (SD). SPSS version 26 (IBM, USA) was used for a one-way ANOVA comparison to check for significant differences between treatments. GraphPad Prism version 8.0 software (GraphPad, San Diego, CA, USA) was used for Student’s *t*-test.

## 3. Results

### 3.1. Putative Sporulation-Responsive Gene Identification and Expression

Among the sixteen well-characterized genes regulating asexual sporulation in *A. nidulans*, seven (namely *flbB*, *flbC*, *flbD*, *veA*, *velB*, *vosA* and *stuA*) had one homolog in the *Fusarium oxysporum* f. sp. *cubense* tropical race 4 genome database. Two or more homologs were present for the *fluG*, *flbA*, *laeA* and *medA* genes. No homolog was found for *brlA*, *flbE* or *velC* (Table 1). Through the BLAST results, we found that two genes, FOIG_01396 and FOIG_07289, share low protein identities with the *abaA* and *wetA* genes, respectively. We further compared these proteins and found that although the entire protein sequence of FOIG_01396 shows low protein identities with AbaA, but much conserved residue existed in the TEA/ATTS domains (Figure S2). Similarly, the entire protein sequences of FOIG_07289 has low protein identity with WetA, but they share much conserved residue inside the ESC/WetA-related domains of them (Figure S2). The TEA/ATTS domain and the ESC/WetA-related domain are predicted as DNA-binding domains. We named the FOIG_01396 as the *abaA*-like (*abaA-L*) gene, and the FOIG_07289 as the *wetA*-like (*wetA-L*) gene.

**Figure 1:**
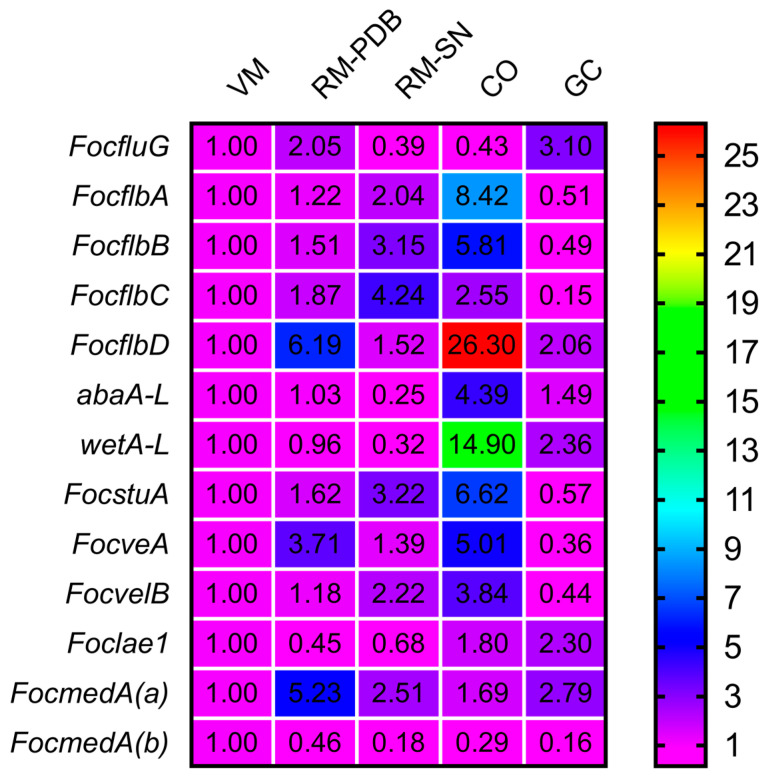
Relative expression levels of thirteen putative sporulation-responsive genes in *Fusarium oxysporum* f. sp. *cubense* strain 58 under vegetative mycelia (VM), microconidia (RM-PDB), macroconidia and chlamydospores formation (RM-SN), conidia (CO, produced in SNA) and germinated conidia (GC) developmental stages. The gene expressions at the different stages were compared with those at the VM stage. Data represent means of three replicates. For gene definitions, see Table 1.

To gain insights into the biological roles of the thirteen putative sporulation-responsive genes in Foc, we used qRT-PCR to examine their relative expression levels at different developmental stages of the fungus, including microconidia (RM-PDB), macroconidium and chlamydospore formation (RM-SN), conidia (CO), germinated conidia (GC) and vegetative mycelia (VM). The relative abundances of the gene transcripts at the RM-PDB, RM-SN, CO and GC stages were compared with their abundances at the VM stage. At the RM-PDB stage, *FocflbD*, *FocveA* and *FocmedA(a)* were significantly upregulated, while *Foclae1* and *FocmedA(b)* were significantly downregulated (Figure 1). At the RM-SN stage, the relative expression levels of *FocflbB*, *FocflbC*, *FocstuA*, *FocvelB* and *FocmedA(a)* were upregulated, while the relative expression levels of *FocfluG*, *abaA-L*, *wetA-L*, *Foclae1* and *FocmedA(b)* were downregulated (Figure 1). At the CO stage, *FocfluG* and *FocmedA(b)* were significantly downregulated, while the other 11 genes were upregulated (Figure 1). At the GC stage, *FocfluG*, *FocflbD*, *wetA-L*, *Foclae1* and *FocmedA(a)* were found to be upregulated, while *FocflbA*, *FocflbB*, *FocflbC*, *FocstuA*, *FocveA*, *FocvelB* and *FocmedA(b)* were downregulated (Figure 1). Taken together, these results suggest that the putative sporulation-responsive genes exhibit different expression patterns at different developmental stages of Foc, and the genes are largely differentially expressed during conidiation and conidia germination in Foc.

To assess the biofunctions of these genes, single-gene deletion mutants were obtained from the wild-type Foc strain 58 using a homologous recombination strategy, and they were hygromycin-resistant. The single-gene deletion was confirmed via PCR using the primer pairs (Table S1) and a Southern blot analysis (Figure S3).

### 3.2. FocstuA, FocveA and FocvelB Are Required for Vegetative Growth in Foc

**Figure 2:**
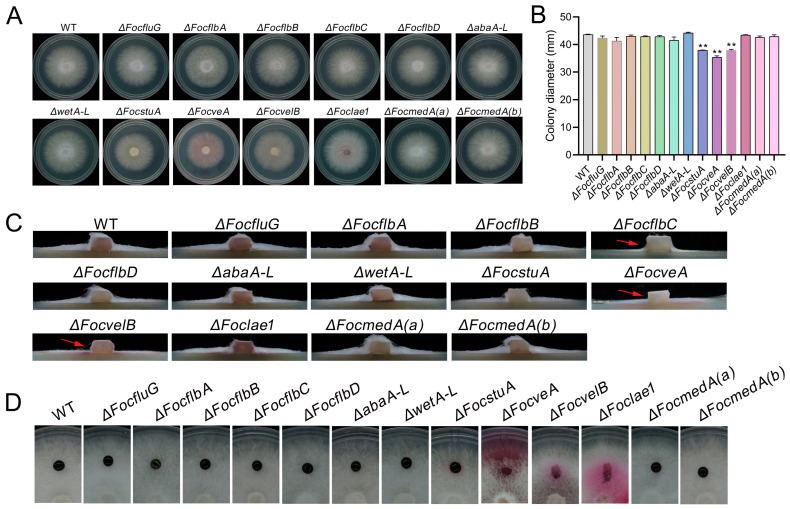
Vegetative growth (**A**,**B**), aerial hypha formation (**C**) and hydrophobicity assays of the aerial hyphae (**D**) of *Fusarium oxysporum* f. sp. *cubense* strain 58 (WT) and the thirteen mutants. Sections (**A**–**C**) show the colony morphology, colony diameter and the transverse section of aerial mycelia after 3 days of incubation at 26 °C on PDA, respectively. The arrow means a significant decrease in aerial hypha formation. In section (**D**), WT and the thirteen mutants were grown for 3 days on CM at 26 °C, with about 30 μL of 1% acid fuchsin (*w*/*v*) applied to the surface of each colony, incubated for 10 min and photographed. The red arrow indicates the decrease in aerial hyphae formation of the mutant strain. Values are presented as means ± SD. Values with double asterisks are significantly different according to Student’s *t*-test at *p* < 0.01.

To analyze the roles of the conidiation-responsive genes in the vegetative growth of Foc, wild-type Foc strain 58 and mutants were grown on PDA media. The results demonstrate that ten out of the thirteen mutants had similar vegetative growths to the wild-type; however, the vegetative growth of the Δ*FocstuA*, Δ*FocveA* and Δ*FocvelB* mutants was retarded compared to that of the WT (Figure 2A,B). Furthermore, like the WT, the aerial hyphae of most of the mutants were not pigmented when grown on PDA, with the exception of those of Δ*FocveA*, Δ*FocvelB* and Δ*Foclae1* mutants, which formed rosy pink colonies (Figure 2A). Moreover, we found that Δ*FocflbC*, Δ*FocveA and* Δ*FocvelB* showed a significant decrease in aerial hypha formation compared to the wild-type and the other mutants (Figure 2C). In addition, we further assessed the hyphal hydrophobicity of the strains and found that the aqueous solution applied to the *velvet*-related mutants Δ*FocveA*, Δ*FocvelB* and Δ*Foclae1* immediately spread onto the surface of the hyphae, while the droplets applied to the wild-type and the other mutants maintained a spherical droplet form, suggesting that the *velvet* genes and *Foclae1* could be involved in mediating hyphal hydrophobicity in Foc (Figure 2D). Collectively, these data suggest that *FocstuA*, *FocveA* and *FocvelB* play important roles in the regulation of the vegetative growth and hyphal development of Foc.

### 3.3. abaA-L Is Essential for Microconidia and Phialides Production in Foc

**Figure 3:**
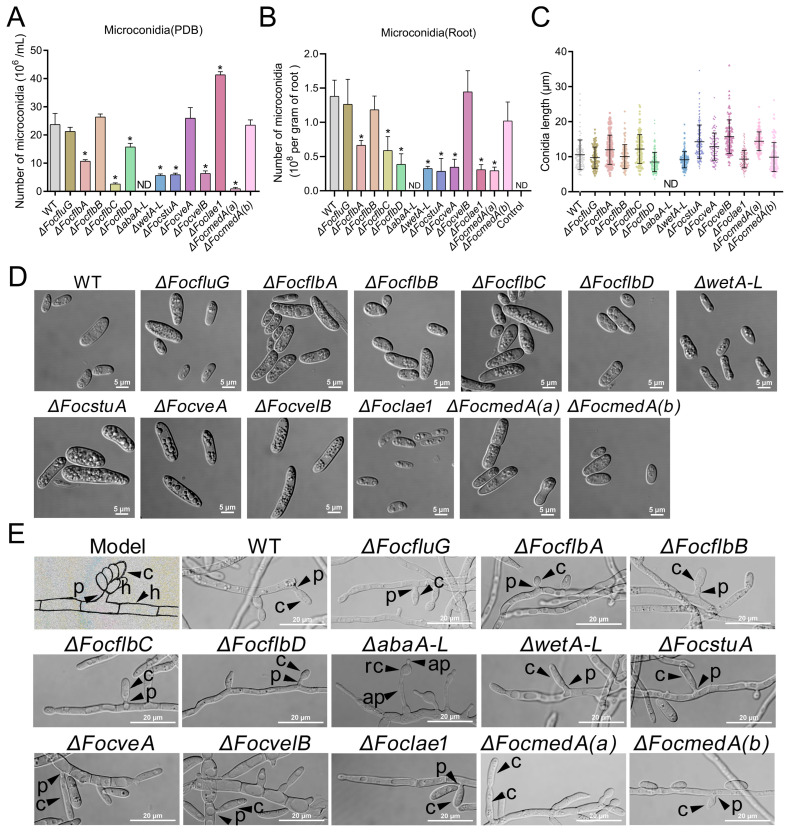
Roles of the sporulation-responsive genes in microconidia production, spore morphology and formation of sporulation structure in *Fusarium oxysporum* f. sp. *cubense* strain 58. Microconidia production in PDB (**A**) and detached banana roots (**B**). Values are presented as means ± SD. Values with asterisks are significantly different according to Student’s *t*-test at *p* < 0.05. ND means no microconidia production. (**C**) Microconidia (produced in PDB) size. Values are presented as means ± SD. Morphology of microconidia (produced in PDB) (**D**) and conidiation structures in PDB (**E**): h, hyphae; p, phialides; c, microconidia; ap, abnormal phialides; rc, round-shaped cell.

A previous study reported that the type of media used to culture Foc determines the type of spores produced by the fungus [62]. The study revealed that microconidia are specifically produced on media with a high carbon source (such as PDB), whereas macroconidia and chlamydospores production are induced on media with a low carbon source (such as SNA). Therefore, we investigated the spore-forming abilities of the various mutants on appropriate media. Our results revealed that the number of microconidia produced by the Δ*FocflbA*, Δ*FocflbC*, Δ*FocflbD*, Δ*abaA-L*, Δ*wetA-L*, Δ*FocstuA*, Δ*FocvelB* and Δ*FocmedA(a)* mutants was significantly less than that produced by the WT in PDB (Figure 3A). Remarkably, microconidiation was completely abolished in the Δ*abaA-L* mutant. However, there was a significant increase in microconidia production in the Δ*Foclae1* mutant compared to the WT (Figure 3A). These results suggest that *abaA-L* is critically important for microconidia production and functions antagonistically to *Foclae1* during microconidiation in Foc.

We similarly determined the number of microconidia produced by the mutants in planta. Consistently, the results showed that the number of microspores produced by Δ*FocflbA*, Δ*FocflbC*, Δ*FocflbD*, Δ*abaA-L*, Δ*wetA-L* and Δ*FocmedA(a)* in banana roots was much lower than that produced by the wild-type strain (Figure 3B). In agreement with the in vitro assay, we observed that no microspores were produced in the banana petioles inoculated with mycelia from the Δ*abaA-L* mutant (Figure S4). We therefore conclude that *FocflbA*, *FocflbC*, *FocflbD*, *abaA-L*, *wetA-L* and *FocmedA(a)* are required for microconidia production both in vitro and in planta. However, it is worth noting that some genes (*FocveA*, *FocvelB* and *Foclae1*) exhibit different regulatory patterns in conidiation in vitro and in planta (Figure 3A,B).

We further observed the microconidia morphology of each strain and found that seven of the thirteen mutants produced physically normal microconidia like the wild-type strain (Figure 3C,D). However, in comparison with the WT, Δ*FocflbA*, Δ*FocflbC*, Δ*FocstuA*, Δ*FocveA*, Δ*FocvelB* and Δ*FocmedA(a)* produced larger microconidia (Figure 3C,D), suggesting that *FocflbA*, *FocflbC*, *FocstuA*, *FocveA*, *FocvelB* and *FocmedA(a)* negatively mediate microconidia size in Foc.

Microconidia are formed from phialides via basipetal division in *F. oxysporum* [63]. Therefore, we further observed the phialide morphology of each sporulation-responsive gene deletion mutant. The results show that eleven mutants could form normal phialides from hyphae, similar to the WT, but the Δ*abaA-L* and Δ*FocmedA(a)* mutants formed abnormal phialides (Figure 3E). The abnormal phialides from the Δ*abaA-L* mutant released round-shaped cells without any microconidium production. In addition, Δ*FocmedA(a)* also failed to produce phialides, but it could produce rod-shaped microconidia directly from hyphae (Figure 3E), which is consistent with a previous study [12]. These results indicate that *abaA-L* and *FocmedA(a)* are critical for phialide formation in Foc.

### 3.4. FocflbA, FocflbD, abaA-L, wetA-L, FocstuA, FocveA, Foclae1 and FocmedA(a) Are Required for Macroconidia Production and Conidiophores Formation in Foc

**Figure 4:**
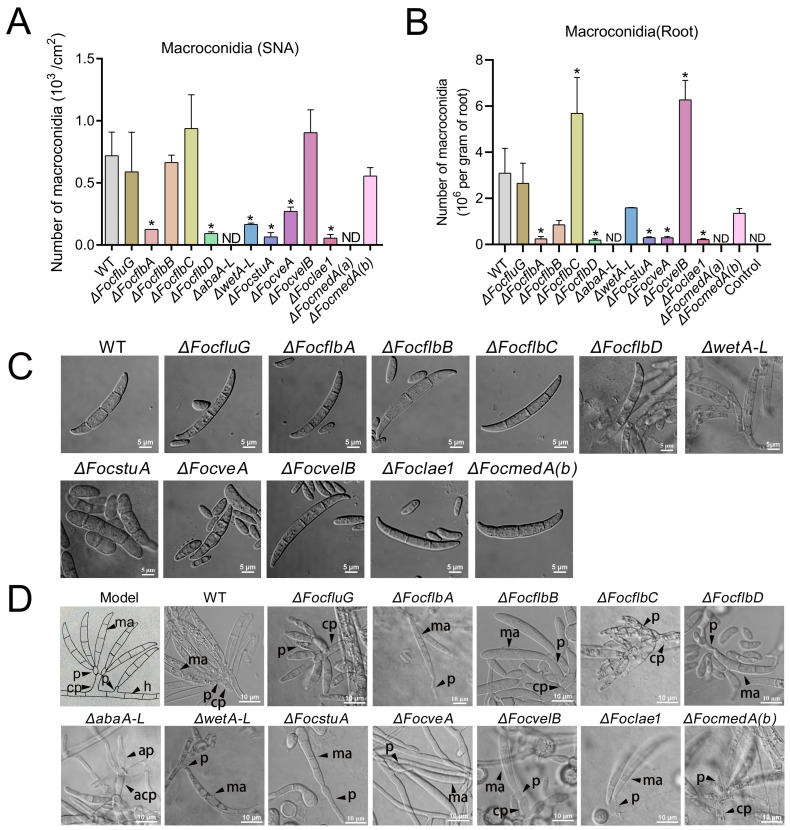
Roles of the sporulation-responsive genes in macroconidium production in Foc. (**A**,**B**) Quantification of macroconidium production on SNA and detached banana roots after 10 days of incubation at 26 °C, respectively. Values are presented as means ± SD. ND means no macroconidia formation. Values with asterisks are significantly different according to Student’s *t*-test at *p* < 0.05. (**C**) Morphology of the macroconidia produced by the various strains (SNA). Images were captured from a scanning confocal microscope. Bar = 5 μm. (**D**) Morphology of conidiophores and phialides (SNA). h, hyphae; ma, macroconidia; p, phialides; ap, abnormal phialides; cp, conidiophore; acp, abnormal conidiophore. Bar = 10 μm.

We found that the quantity of macroconidia produced by the Δ*FocfluG*, Δ*FocflbB*, Δ*FocflbC*, Δ*FocvelB* and Δ*FocmedA(b)* mutants was similar to that produced by the wild-type strain on SNA (Figure 4A). The remaining six mutants (Δ*wetA-L*, Δ*FocflbA*, Δ*FocflbD*, Δ*FocveA*, Δ*Foclae1* and Δ*FocstuA*) produced significantly lower amounts of macroconidia than the wild-type strain on SNA (Figure 4A), suggesting that *wetA-L*, *FocflbA*, *FocflbD*, *FocveA*, *Foclae1* and *FocstuA* play important roles in mediating macroconidia production. Interestingly, the Δ*abaA-L* and Δ*FocmedA(a)* mutants could not produce any macroconidium on SNA (Figure 4A), suggesting that *abaA-L* and *FocmedA(a)* are essential genes for macroconidia production in Foc.

We also quantified the amounts of macroconidia produced by the various fungal strains in planta and found that the Δ*FocflbC* and Δ*FocvelB* mutants produced significantly higher amounts of macroconidia in the detached banana roots than the wild type (Figure 4B). However, the number of macrospores produced by the Δ*FocflbA*, Δ*FocflbD*, Δ*FocstuA*, Δ*FocveA* and Δ*Foclae1* mutants was also much lower than that produced by the wild-type strain (Figure 4B). Consistent with the in vitro assay, we could not find a single macrospore in the banana roots or banana petioles infected with mycelia from the Δ*abaA-L* and Δ*FocmedA(a)* mutants (Figure 4B and Figure S4). Collectively, our data suggest that, of all the sporulation-responsive genes in Foc, *abaA-L*, and *FocmedA(a)* play the most critical roles in macroconidia formation both in vitro and in planta.

We further observed the morphology of the macroconidia of each strain and found that nine gene mutants produced falcate macroconidia similar to those of the wild-type strain (Figure 4C). In contrast, the macroconidia produced by the Δ*FocstuA* mutant were thicker, lacking the pointed ends observed in the wild type (Figure 4C). In addition, when cultured on SNA, the Δ*wetA-L* mutant produced few immature macroconidia with a seemingly empty cytoplasm (Figure 4C). Thus, *wetA-L* and *FocstuA* could be involved in ensuring macroconidia maturation and normal morphology in Foc, respectively.

In *F. oxysporum*, macroconidia are usually produced from phialides arising from conidiophores but not from those arising directly from vegetative hyphae [12,44]. Therefore, we further investigated the production and morphology of the conidiophores in the wild-type and mutant strains. We noticed that the Δ*FocfluG*, Δ*FocflbB*, Δ*FocflbC*, Δ*FocvelB* and Δ*FocmedA(b)* mutants produced conidiophores that were morphologically similar to those of the wild-type (Figure 4D). However, no single conidiophore was produced by the Δ*FocflbA*, Δ*FocflbD*, Δ*wetA-L*, Δ*FocstuA*, Δ*FocveA*, Δ*Foclae1* and Δ*FocmedA(a)* mutants (Figure 4D). Although the Δ*abaA-L* mutant produced abnormal conidiophores, which gave rise to abnormal phialides, the mutant was obviously lacking macroconidia (Figure 4D). Taken together, these results demonstrate that the *FocflbA*, *FocflbD*, *abaA-L*, *wetA-L*, *FocstuA*, *FocveA*, *Foclae1* and *FocmedA(a)* genes play important roles in the regulation of normal conidiophore formation in Foc.

### 3.5. FocflbB, FocflbC, FocflbD, abaA-L, wetA-L, FocvelB and Foclae1 Control Chlamydosporulation In Vitro and in Planta in Foc

**Figure 5:**
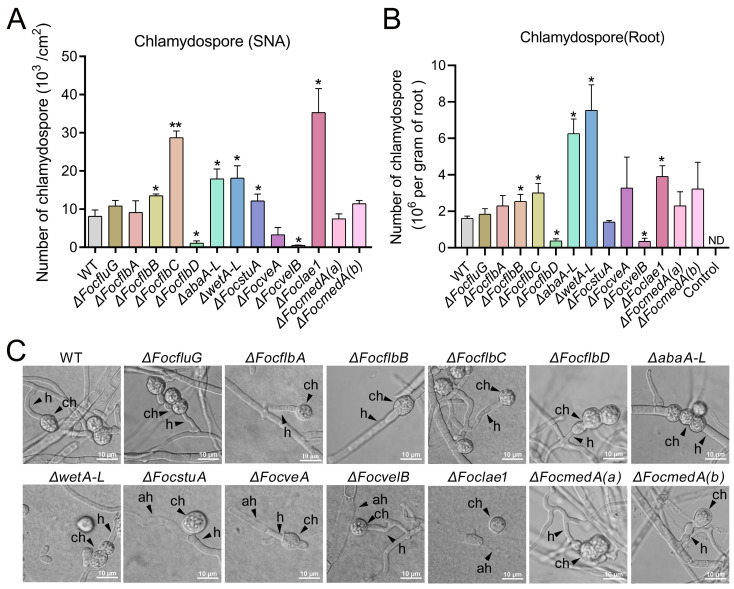
Roles of the sporulation-responsive genes in chlamydospore production, chlamydospore morphology and sporulation process in Foc. (**A**,**B**) Number of chlamydospores produced by the wild-type and mutant strains grown on SNA and detached banana root at 26 °C for 10 days, respectively. Values are presented as means ± SD. ND means no chlamydospore formation. Values with an asterisk or two asterisks are significantly different according to Student’s *t*-test at *p* < 0.05 or *p* < 0.01, respectively. (**C**) Conidiogenesis of chlamydospores on SNA. Like those of the wild-type, the mutants’ globose chlamydospores are produced from modification of hyphae. h, hyphae; ah, autolysis of hyphae; ch, chlamydospore. Bar = 10 μm.

Chlamydospores can survive in soil for a long time and act as inocula in the fungal infection cycle [15]. Our results show that Δ*FocflbB*, Δ*FocflbC*, Δ*abaA-L*, Δ*wetA-L*, Δ*FocstuA* and Δ*Foclae1* produced significantly more chlamydospores than the wild-type strain on SNA (Figure 5A and Figure S5), unlike Δ*FocflbD* and Δ*FocvelB*, which produced significantly less chlamydospores than the WT (Figure 5A). In addition, we further determined the chlamydospore production of the above strains in planta by inoculating their mycelia on banana roots. We found that the number of chlamydospores produced by the Δ*FocflbB*, Δ*FocflbC*, Δ*abaA-L*, Δ*wetA-L* and Δ*Foclae1* mutants in planta was higher than that produced by the WT, whereas the quantity of chlamydospores produced by the Δ*FocflbD* and Δ*FocvelB* mutants was less than that produced by the WT (Figure 5B). These results are consistent with those obtained in vitro (Figure 5A). Furthermore, our morphological analysis of the chlamydospores of all the mutants showed similar phenotypes to those of the wild type, including the development of globose chlamydospores from the fungal hyphae (Figure 5C). Taken together, *FocflbB*, *FocflbC*, *abaA-L*, *wetA-L* and *FocL*a*e1* negatively regulate chlamydospore formation, whereas *FocflbD* and *FocvelB* facilitate Foc chlamydosporulation both in vitro and in planta.

### 3.6. Functional Relationship of the Sporulation-Responsive Genes Regulating Microconidia Production in Foc

It can be deduced from Figure 3A that *FocflbA*, *FocflbC*, *FocflbD*, *abaA-L*, *wetA-L*, *FocstuA*, *FocvelB*, *Foclae1* and *FocmedA(a)* control microconidia production under high-carbon-source conditions, whereas it can be deduced from Figure 4A and Figure 5A that some of these genes also control macroconidia and chlamydospores formation under low-carbon-source conditions. However, the relationship between the regulatory functions of these genes remains unclear in Foc. Thus, we used quantitative real-time RT-PCR (qRT-PCR), yeast one-hybrid (Y1H) and chromatin immunoprecipitation–quantitative polymerase chain reaction (ChIP-qPCR) analyses to clarify this functional relationship among the conidiogenesis-responsive genes in Foc.

**Figure 6:**
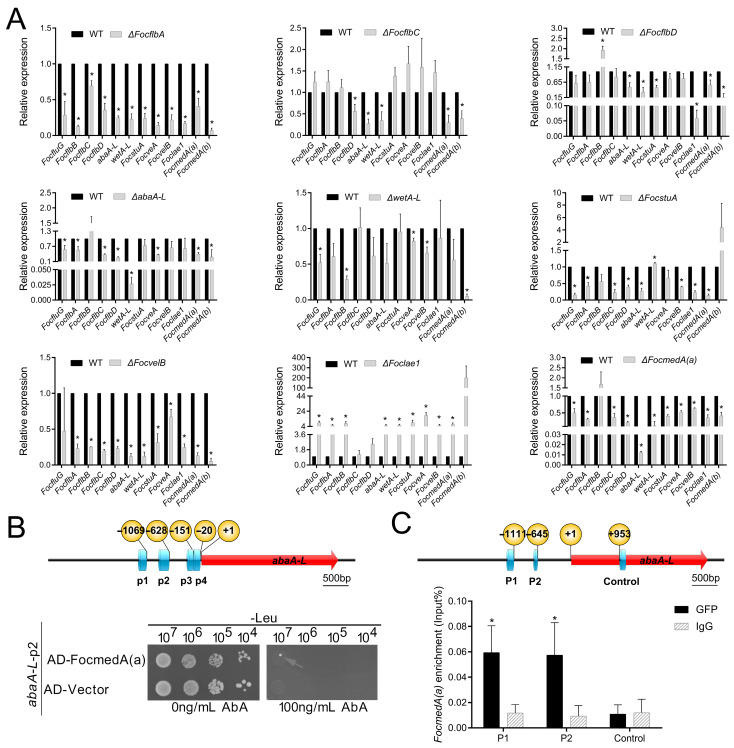
Identification of regulatory relationships among the microconidia sporulation-related genes in Foc. (**A**) The relative expression levels of the sporulation-responsive genes under high-carbon-source conditions. Three independent experiments were performed; each sample for qRT-PCR experiment was run in three technical replicates. (**B**) FocMedA(a) protein interacts with the promoter of *abaA-L* gene as revealed via Y1H assay. (**C**) FocMedA(a) protein binds to the promoter of *abaA-L* gene as revealed via ChIP-qPCR assay. Three independent experiments were performed. Values are presented as means ± SD. Values with asterisks are significantly different according to Student’s *t*-test at *p* < 0.05.

**Figure 7:**
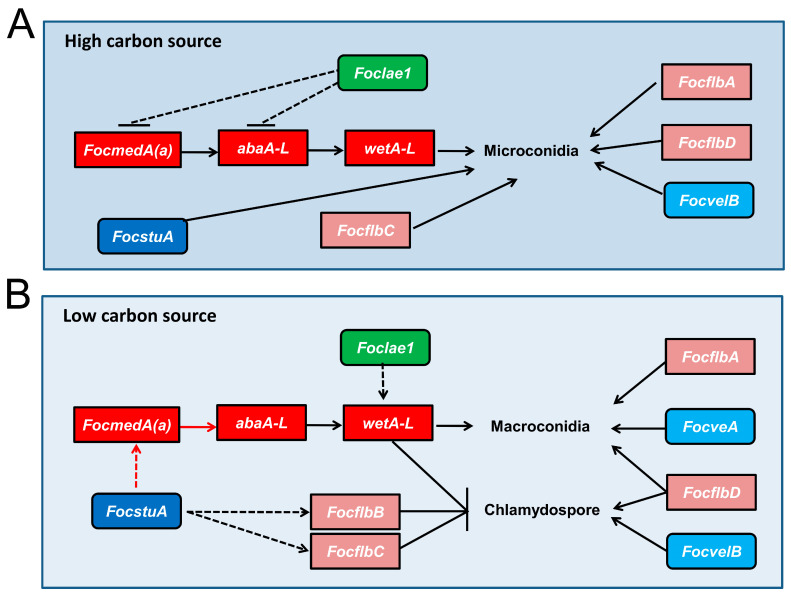
Proposed working model for the regulatory network of the sporulation-responsive genes in *Fusarium oxysporum* f. sp. *cubense* (Foc). (**A**) Schematic diagram showing the regulatory network of microconidiation-related genes under high-carbon-source conditions in Foc. (**B**) Schematic diagram showing the regulatory network of macroconidiation- and chlamydospore-related genes under low-carbon-source conditions in Foc. Black solid arrows indicate promotion of gene expression or conidiation; vertical/horizontal blunt lines show repression of gene expression or conidiation. Solid straight lines mean the regulation relationship was confirmed, whereas dashed straight lines mean the regulation relationship was not confirmed. Red dashed and solid arrows indicate that the regulatory relationships are not fit for chlamydospore formation, but they are fit for regulation of macroconidia formation in Foc.

In the *Aspergillus* genus, *brlA*-*abaA*-*wetA* forms the central regulatory pathway (CRP) that promotes successful conidiophore development and spore maturation [20,28,30,31]. In Foc, the biofunctions of *abaA-L* and *wetA-L* are very similar to the *abaA* and *wetA* in *Aspergillus*, but there is no homolog of *brlA* in Foc (Table 1); therefore, we hypothesized that there is/are other gene(s) that may play the role played by *brlA* in *Aspergillus.* Based on our earlier results, *FocmedA(a)* was found to be essential for phialide and conidiophore formation during conidiation (Figure 3E and Figure 4D), and our qRT-PCR data revealed that the expression level of *abaA-L* was significantly reduced by 0.01-fold in the Δ*FocmedA(a)* mutant when compared to that in the wild-type strain (Figure 6A). We therefore reasoned that the expression of *abaA-L* could be activated by *FocmedA(a)*. We tested this hypothesis using Y1H and ChIP-qPCR assays (Figure 6B,C). Δ*FocmedA(a)-C* was used for ChIP -qPCR assay, which showed similar phenotypes with the wild type strains (Figure S6). Following the Y1H assay, we identified four binding motifs (p1, p2, p3 and p4) on the promoter region of *abaA-L*, and we found that three of them (p1, p3 and p4) were autoactivated with the pGADT7 vector (Figure S1). The other motif p2 was not autoactivated and could interact with FocMedA(a) (Figure 6B), suggesting that FocMedA(a) can bind the promoter of *abaA-L.* Furthermore, our ChIP-qPCR analysis further confirmed that FocMedA(a) directly binds *abaA-L* to activate its gene expression (Figure 6C). In addition, the transcript abundance of *wetA-L* was significantly reduced by 0.03-fold in the Δ*abaA-L* mutant (Figure 6A), implying that *abaA-L* could directly regulate the expression of *wetA-L* in Foc (Figure 7A).

Initiation of the asexual development pathway in *A. nidulans* is regulated by upstream developmental activators (UDAs), including *fluG*, *flbA*, *flbB*, *flbC*, *flbD* and *flbE*, and deletion of any one of these genes results in a greatly reduced expression of the downstream CRP gene *brlA*, causing defects in phialides and conidiophores formation and conidia production [21]. However, our results show that there was no homolog of the *FlbE* gene in the Foc genome (Table 1), and deletion of *FocfluG* or *FocflbB* did not affect conidia production (Figure 3A,B). In addition, deletion of *FocflbA*, *FocflbC* or *FocflbD* resulted in reduced microconidia production but did not affect the formation of phialides (Figure 3E), suggesting that *FocflbA*, *FocflbC* and *FocflbD* do not function as upstream components of the *FocmedA(a)*, *abaA-L* and *wetA-L* genes. We further found that the expression levels of the *FocmedA(a)*, *abaA-L* and *wetA-L* genes were reduced by 44.3–78.3% in the Δ*FocflbA*, Δ*FocflbC* and Δ*FocflbD* mutants (Figure 6A), and we speculated that the *FocflbA*, *FocflbC* and *FocflbD* genes may be indirectly involved in the regulation of the *FocmedA(a)*, *abaA-L* and *wetA-L* genes (Figure 7A). These results simply mean that the homologs of the UDA genes in Foc perform different roles from those in *A. nidulans*.

The other proteins (StuA, Velvet complex and LaeA) were also shown to influence conidia formation in *A. nidulans* [27]. In Foc, deletion of *FocstuA* and *FocvelB* led to reduce microconidia production but did not influence phialides formation (Figure 3E). Although we found that the expression levels of *FocmedA(a)* and *abaA-L* were significantly reduced in the *FocstuA* and *FocvelB* deletion mutants (Figure 6A), we still thought that *FocstuA* and *FocvelB* may indirectly influence *FocmedA(a)* and *abaA-L* expression. In contrast, the Δ*Foclae1* mutant produced significantly more microconidia than the wild-type strain, and the expression levels of *FocmedA(a)* and *abaA-L* were 5 times higher than those of the wild-type strain (Figure 6A), indicating that *Foclae1* may repress *FocmedA(a)* and *abaA-L* expression (Figure 7A).

### 3.7. Functional Relationship of the Sporulation-Responsive Genes Regulating Macroconidia and Chlamydospores Production in Foc

**Figure 8:**
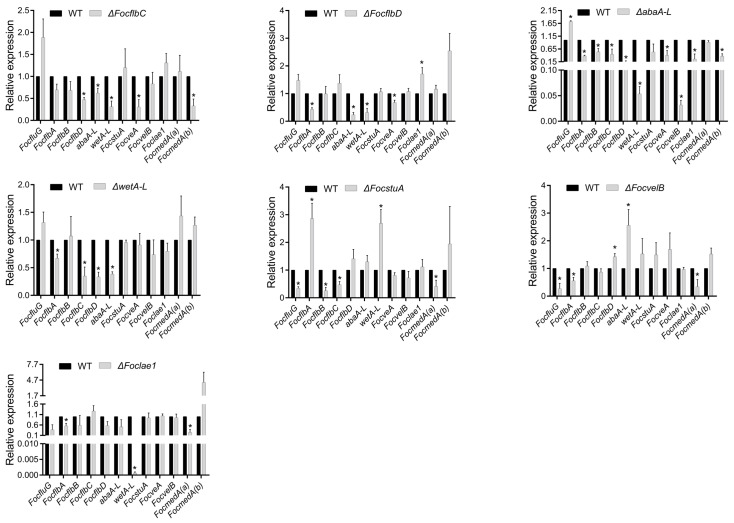
Identification of regulatory relationships among the sporulation-related genes of Foc under low-carbon-source conditions (liquid Spezieller Nährstoffarmer medium). Three independent experiments were performed. Values are presented as means ± SD. Values with asterisks are significantly different according to Student’s *t*-test at *p* < 0.05.

We demonstrated earlier that six genes, namely *FocflbA*, *FocflbD*, *abaA-L*, *wetA-L*, *FocstuA* and *FocmedA(a)*, positively regulate both microconidia and macroconidia formation in Foc (Figure 3A and Figure 4A), and that *FocmedA(a)*, *abaA-L* and *wetA-L* may work as a cascade in the regulation of microconidia production. During the macroconidiation process, *FocmedA(a) and abaA-L* are essential for the formation of phialides, conidiophores and macroconidia, and *wetA-L* controls macroconidia maturity (Figure 4), suggesting that these three genes also play important roles in the macroconidia production process (Figure 7B). *FocstuA* was shown to be required for the formation of conidiophores (Figure 4D). Our qRT-PCR revealed that the expression level of *FocmedA(a)* in the Δ*FocstuA* mutant was significantly downregulated (Figure 8), suggesting that *FocstuA* may directly activate *FocmedA(a)* expression. Although *FocflbD* is also essential for the formation of conidiophores, the deletion of *FocflbD* does not affect the expression of *FocmedA(a)* (Figure 8). In addition, although *Foclae1* serves as a negative regulator of microconidia formation under PDB conditions (Figure 3A), it positively regulates macroconidia formation (Figure 4A). We observed that the expression level of *wetA-L* was decreased by 99.9% in the *Foclae1* deletion mutant (Figure 8), implying that *Foclae1* may directly control *wetA-L* expression (Figure 7B).

Under SN conditions, *abaA-L*, *wetA-L* and *Foclae1* played positive roles in the regulation of macroconidia formation (Figure 4A), but their deletion mutants showed increased chlamydospores production (Figure 5A), suggesting that they act as negative regulators of chlamydospore formation (Figure 7B). Based on the chlamydospore production assay (Figure 5), we found that *FocflbB*, *FocflbC* and *FocstuA* suppressed chlamydospore formation, whereas *FocflbD* and *FocvelB* promoted chlamydospore formation in Foc (Figure 7B). The qRT-PCR results showed that the expression of the *FocflbB* and *FocflbC* genes was significantly downregulated in Δ*FocstuA* compared to that in the wild-type strain (Figure 8), indicating that *FocstuA* may be located upstream of *FocflbB* and *FocflbC*, and that they synergistically inhibit chlamydospore formation in Foc (Figure 7B).

### 3.8. FocfluG, FocflbA, FocflbC, FocflbD, FocstuA, FocveA, FocvelB and Foclae1 Are Required for the Pathogenicity of Foc

**Figure 9:**
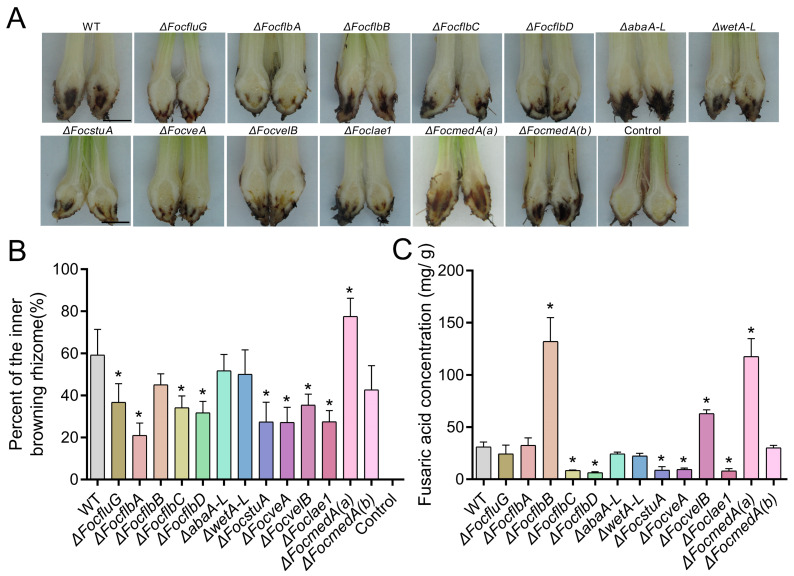
Roles of the putative sporulation-responsive genes in the pathogenicity of Foc. (**A**) Disease symptoms on corms of banana plantlets infected with conidia suspensions from the indicated strains. Scale bar = 1 cm. (**B**) Percentage of inner necrosis on the infected banana corms. (**C**) Quantification of fusaric acid (FA) produced by the different fungal strains under liquid Czapek–Dox medium. FA production levels are presented in mg/g dry mycelial weight. Three independent experiments were performed. Values are presented as means ± SD. Values with asterisks are significantly different according to Student’s *t*-test at *p* < 0.05.

We explored the pathogenicity of the various strains in banana roots. As shown in Figure 9A, the severity of the browning of vascular tissues in the banana plants infected with Δ*FocflbB*, Δ*abaA-L*, Δ*wetA-L* and Δ*FocmedA(b)* mutants was similar to that of the banana plants infected with the wild-type strain, though the disease symptom caused by Δ*FocmedA(a)* appeared to be much more severe. In contrast, the severity of corm browning symptoms in the banana plants infected with Δ*FocfluG*, Δ*FocflbA*, Δ*FocflbC*, Δ*FocflbD*, Δ*FocstuA*, Δ*FocveA*, Δ*FocvelB* and Δ*Foclae1* mutants was much less than that in the banana plants infected with the wild-type strain (Figure 9A). At 60 days post-inoculation (dpi), the average percentage of the inner browning rhizome of the plant infected with the wild-type strain was 59.3%, but those of the plants infected with Δ*FocfluG*, Δ*FocflbA*, Δ*FocflbC*, Δ*FocflbD*, Δ*FocstuA*, Δ*FocveA*, Δ*FocvelB* and Δ*Foclae1* mutants were only 36.8%, 21.0%, 34.2%, 31.8%, 27.5%, 27.2%, 35.4% and 27.5% (Figure 9B), respectively. These results indicate that the *FocfluG*, *FocflbA*, *FocflbC*, *FocflbD*, *FocstuA*, *FocveA*, *FocvelB* and *Foclae1* genes are positive regulators of pathogenicity in Foc.

Next, we examined whether these genes play any role in the penetration ability of the fungus, but our results show that all the tested mutant strains can successfully penetrate cellophane membranes similar to the wild-type strain (Figure S7). Fusaric acid (FA) production by Foc is associated with pathogenicity in banana plants [64]. In comparison with the WT, FA production was found to be significantly reduced in Δ*FocflbC*, Δ*FocflbD*, Δ*FocstuA*, Δ*FocveA* and Δ*Foclae1* mutants, but significantly increased in the Δ*FocmedA(a)* mutant (Figure 9C). These results are consistent with the results presented in Figure 9A,B. However, contrary to our observations in Figure 9A,B, the FA production in the Δ*FocflbB* and Δ*FocvelB* mutants was observed to significantly increase, indicating that *FocflbB* and *FocvelB* may be regulators of other virulence factors in Foc.

## 4. Discussion

**Figure 10:**
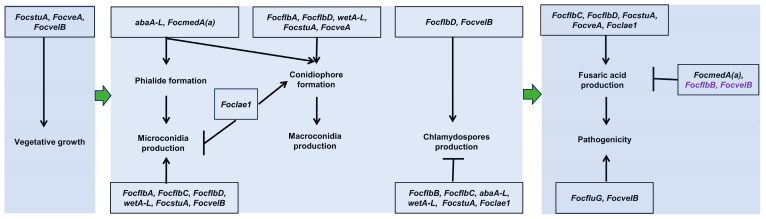
A summary of the biofunctions of the genes analyzed in this study. Black solid arrows indicate promotion of vegetative growth, conidiation, fusaric acid production or pathogenicity; vertical/horizontal blunt lines show repression conidiation or fusaric acid production. *FocflbB* and *FocvelB* marked in purple indicate that these genes negatively regulate the formation of fusaric acid in Foc, but their disruption mutants do not result in a significant increase in pathogenicity to bananas.

To date, a sexual reproduction process has not been found in Foc, so three kinds of asexual spores play important roles in the fungal survival, the infection cycle and the spread process. In this study, we characterized the biofunctions of 13 genes in the life cycle of Foc and systemically established their roles and relationships underlying asexual reproduction (Figure 7 and Figure 10).

In *A. nidulans*, the central regulatory pathway (CRP) involves the three genes *brlA*, *abaA* and *wetA*, and it is of central importance in controlling conidiophore and phialide formation and conidia maturation [27]. *brlA* mediates the morphological switch from the vegetative growth pattern of hyphal cells to the budding growth pattern of conidiophores, and it also activates downstream *abaA* gene expression [65]. *abaA* is responsible for the development of conidiophores and phialides, and it also activates downstream *wetA* gene expression [65]. *wetA* is responsible for conidia maturation [65]. However, *brlA* homologs are present only in *Aspergillus*, *Neosartorya*, *Penicillium* and *Talaromyces* genera [27]. In this study, we found that the functions of *abaA-L* and *wetA-L* in Foc appear to be similar to *abaA* and *wetA* in *A*. *nidulans* (Figure 3 and Figure 4). The expression of *wetA-L* was significantly downregulated in the Δ*abaA-L* mutant (Figure 6A and Figure 8), indicating that *abaA-L* may directly regulate *wetA-L* expression.

*brlA* is the initial CRP gene in *A. nidulans*, but its homolog gene was not identified in Foc; we wondered which gene in Foc plays the role of *brlA* in *A. nidulans* (especially in the activation of *abaA*). We found that *FocmedA(a)* was essential for phialide and conidiophore formation, which is similar to the functions of *abaA-L* (Figure 3E and Figure 4D), and the expression of *FocmedA(a)* was significantly upregulated during microconidiation and macroconidiation (Figure 1). Moreover, the relative expression of *abaA-L* was decreased by 99.7% in the Δ*FocmedA(a)* mutant (Figure 6A), and the results of our Y1H assay and ChIP-qPCR analysis further confirmed that FocMedA(a) could bind the promoter region of *abaA-L* (Figure 6B,C). These results indicate that *FocmedA(a)* functions as an upstream activator of *abaA-L* in Foc (Figure 7). The homologs of *FocmedA(a)* have been identified in some other filamentous fungi. In *F. graminearum*, loss of the *medA* homolog gene *FgMed1* affects the formation of phialide, but the *FgMed1* deletion mutant can still produce conidia directly from abnormal conidiophores [66]. In *A. nidulans*, loss of *medA* causes proliferation of branching chains of metulae and delays phialide formation and conidia production; it also affects the expression of *brlA* and *abaA* [40,65]. In *A. fumigatus*, loss of *medA* does not affect conidiophore formation but tampers with the construction of phialides, and *medA* does not regulate the expression of *brlA* and *abaA* [67]. The above results suggest that *medA* homologs share important but species-specific roles in the regulation of conidiophore or phialide formation in filamentous fungi.

In *A. nidulans*, activation of CRP pathway depends on the expression of the various “fluffy” genes, including *fluG*, *flbA*, *flbB*, *flbC*, *flbD* and *flbE*, which belong to the upstream developmental activators (UDAs) [27]. Mutants of any of the above six genes produce massive aerial hyphae, resulting in the formation of cotton-like colonies with a “fluffy” morphology, and have defects in *brlA* expression and conidia production [27]. However, in this study, we found no homolog of *flbE* in Foc (Table 1), and deletion of other homologs of the *A. nidulans* “fluffy” genes (including *FocfluG*, *FocflbA*, *FocflbB* and *FocflbD*), except for *FocflbC*, did not affect the colony morphology and the formation of phialides. Our results suggest that the “fluffy” genes in Foc showed significant functional differences from their homologs in *A. nidulans*. In *F. graminearum*, *FgfluG*, *FgflbB* and *FgflbE* are also dispensable for conidiation, whereas loss of *FgflbC* or *FgflbD* leads to reduction in conidia production or completely abolishes conidiation, respectively [68]. In *F. graminearum*, the mutant of the *FgflbD* gene also forms the usual fluffy colony, does not produce any sporulation structure and significantly downregulates the expression of *FgabaA* [68], suggesting that some “fluffy” genes play different roles in *Fusarium* species.

*stuA* is not required for vegetative growth in *A. nidulans*, but it negatively regulates *brlA* and *abaA* expressions, and *StuA* disruption mutants have abnormal conidial apparatus (short conidiophores) [40,69,70]. Herein, although we found that *FocstuA* is not necessary for phialide formation (Figure 3E), it is, however, necessary for vegetative growth, microconidia and macroconidia production, and conidiophore formation (Figure 2, Figure 3A and Figure 4). Additionally, Δ*FocstuA* significantly downregulates *FocmedA(a)* but not *abaA-L* expressions (Figure 8). Moreover, the defects observed in the Δ*FocstuA* mutant in macroconidia production and conidiophore formation are similar to those observed in Δ*FocmedA(a)* (Figure 4), indicating that *FocmedA(a)* may function downstream of *FocstuA* (Figure 7B). In three other filamentous fungi, namely *F. graminearum*, *Aspergillus fumigatus* and *Penicillium marneffei*, deletion of *stuA* homolog genes also affects the formation of sporulation structures [71,72,73]. In *F. graminearum*, Δ*FgstuA* lacks conidiophores and phialides, thereby forming few aberrant macroconidia [71]. In *Aspergillus fumigatus*, *AfstuA* deletion mutants produce abnormally short or no conidiophores [72]. In *Penicillium marneffei*, *PmstuA* is necessary for metula and phialide formation [73]. These suggest that *stuA* homologs have conserved roles in conidiation, but their regulatory relationships with other conidiation regulators in these three fungi have not been reported [71,72,73].

Under low-carbon or nutrient-deficient conditions, a large number of macroconidia and chlamydospores and a small number of microconidia are produced by Foc, but chlamydospores are directly formed from hyphae, and this process does not require the formation of special conidiation structures [9,74]. In this study, we found that some genes play opposite roles in conidia and chlamydospores production processes in Foc. We observed that *FocmedA(a)* is critical for microconidia and macroconidia production but dispensable for chlamydospore formation (Figure 3, Figure 4 and Figure 5), which is consistent with a previous report [12]. However, *FocflbB* is not required for conidia production but negatively regulates chlamydospore production (Figure 3 and Figure 5). *abaA-L*, *wetA-L* and *Foclae1* were found to positively regulate conidia production (except for *Foclae1*) but negatively regulate chlamydospore formation (Figure 3 and Figure 5). These results suggest that, in some conditions, Foc can produce conidia and chlamydospores at the same time, but the regulatory pathways between chlamydospores and conidia production are separate (Figure 7).

Of all the genes studied herein, only *FocstuA*, *FocveA* and *FocvelB* are involved in regulating vegetative growth in Foc (Figure 2). In addition to regulating vegetative growth, *FocstuA*, *FocveA* and *FocvelB* also control the fungal virulence and the production of fusaric acid mycotoxin (Figure 9). However, *abaA-L* null mutants could not produce micro- or macroconidia (Figure 3 and Figure 4), in addition to having no impact on fungal virulence (Figure 9A,B). Furthermore, the Δ*FocmedA(a)* mutant had significantly reduced number of microconidia or the macroconidia production may be completely abolished (Figure 3A and Figure 4A), but it displayed increased virulence (Figure 9A,B). Based on these results, it seems that conidia formation during the infection process is not essential (except for chlamydospore production) for the pathogenicity of Foc.

In summary, the present study systematically characterized the biofunctions of sporulation-responsive genes in Foc (Figure 10). Their roles in the formation of microconidia, macroconidia, chlamydospores, phialides and conidiophore were established (Figure 10). We also unveiled their relationship with the fungal virulence and fusaric acid biosynthesis (Figure 10). Importantly, this study revealed the interdependence of these genes in terms of their functions in regulating asexual reproduction in Foc (Figure 7). However, some unknown species-specific regulators seem to exist; further studies are therefore required to uncover such regulators.

## Figures and Tables

**Table 1:** Homologs of sporulation-related genes of *Aspergillus nidulans* in *Fusarium oxysporum* f. sp. *cubense*.

*Aspergillus nidulans*			*Fusarium oxysporum* f. sp. *cubense*					
Gene	Protein Accession Number ^a^	Domain ^b^	Number of Homologs ^c^	Homologs ^c^	Max Score (e-Value) ^c^	Query Coverage ^c^	Percent Identity ^c^	Function
*fluG*	P38094	Glutamine synthetase, catalytic domain; amidohydrolase	2	FOIG_07155	612 (0.0)	99%	41%	Present work (*FocfluG*)
				FOIG_11529	182 (6 × 10 ^−50^)	50%	29%	Unknown
*flbA*	P38093	Regulator of G protein signaling domain; domain found in Dishevelled, Egl-10 and Pleckstrin (DEP)	3	FOIG_08170	607 (0.0)	72%	60%	Present work (*FocflbA*)
				FOIG_16270	454 (2 × 10 ^−153^)	67%	49%	Unknown
				FOIG_13410	309 (3 × 10 ^−112^)	65%	44%	Unknown
*flbB*	C8VBM8	bZIP transcription factor	1	FOIG_00530	205 (2 × 10 ^−62^)	90%	36%	Present work (*FocflbB*)
*flbC*	G5EAS8	Zinc finger, C2H2 type	1	FOIG_03036	217 (5 × 10 ^−68^)	66%	56%	Present work (*FocflbC*)
*flbD*	G5EAY5	Myb-like DNA-binding domain	1	FOIG_06682	142 (9 × 10 ^−41^)	33%	65%	Present work (*FocflbD*)
*flbE*	Q5BFF9	No	0	-	-	-	-	-
*brlA*	P10069	Zinc-finger double domain	0	-	-	-	-	-
*abaA*	P20945	TEA/ATTS domain	1	FOIG_01396	67.4 (2 × 10 ^−11^)	10%	41%	Present work (*abaA-L*)
*wetA*	P22022	ESC1/WetA-related domain	1	FOIG_07289	61.6 (3 × 10 ^−10^)	10%	68%	Present work (*wetA-L*)
*stuA*	P36011	KilA-N domain	1	FOIG_07247	249 (7 × 10 ^−75^)	33%	61%	Present work (*FocstuA*)
*veA*	C8VTV4	Velvet factor	1	FOIG_00370	221 (1 × 10 ^−76^)	49%	48%	Present work (*FocveA*)
*velB*	C8VTS4	Velvet factor	1	FOIG_00471	170 (2 × 10 ^−89^)	83%	50%	Present work (*FocvelB*)
*velC*	Q5BBM1	Velvet factor	0	-	-	-	-	-
*vosA*	Q5BBX1	Velvet factor	1	FOIG_05756	111 (8 × 10 ^−27^)	38%	37%	Unknown
*laeA*	C8VQG9	Methyltransferase domain	6	FOIG_01530	180 (3 × 10 ^−53^)	75%	35%	Present work (*Foclae1*)
				FOIG_10149	188 (8 × 10 ^−57^)	77%	37%	Unknown
				FOIG_15419	191 (9 × 10 ^−58^)	81%	36%	Unknown
				FOIG_16515	181 (4 × 10 ^−54^)	77%	36%	Unknown
				FOIG_10023	181 (1 × 10 ^−53^)	76%	33%	Unknown
				FOIG_11569	179 (2 × 10 ^−53^)	77%	34%	Unknown
*medA*	O74251	No	2	FOIG_10811	292 (2 × 10 ^−91^)	72%	41%	Present work (*FocmedA(a)*)
				FOIG_16330	254 (2 × 10 ^−80^)	58%	42%	Present work (*FocmedA(b)*)

Notes: ^a^ and ^b^, the protein accession numbers and protein domains of *Aspergillus nidulans* were collected from the UniProt database (https://www.uniprot.org/uniprot/, accessed on 1 December 2021) and Pfam version 35.0 (https://pfam.xfam.org/, accessed on 1 December 2021), respectively. ^c^, the total number of homologs, protein alignment score, e-values (numbers in parenthesis), query coverage and percent identity in Foc were collected from NCBI using BLASTp method. “-” indicates that no homolog gene exists or no data.

## Data Availability

Data are contained within the article and Supplementary Materials.

## Supplementary Materials

The following supporting information can be downloaded at https://www.mdpi.com/article/10.3390/jof10010001/s1, Figure S1: Y1H technology was used to determine whether FocMedA(a) proteins interact with *abaA-L* promoter; Figure S2: Similarity analysis between AbaA and AbaA-L, WetA and WetA-L; Figure S3: Southern blot hybridization analyses for mutants of the gene deletion mutants of Foc obtained from this study; Figure S4: Microconidia and macroconidia productions on banana petioles for the wild-type strain and the sporulation-related gene deletion mutants; Figure S5: Chlamydospore production ability of the various mutants. Photographs were taken at the 10th day of incubation; Figure S6: Roles of the *FocmedA(a)* gene in microconidia and macroconidia production, fusaric acid production and pathogenicity in *Fusarium oxysporum* f. sp. *cubense*. Figure S7: Conidiation-related genes are not required for cellophane membrane penetration in Foc; Table S1: The primers used for gene disruption and mutant identification; Table S2: The primers used for qRT-PCR and vector construction; Table S3: The strains used in this study.
