# Supplementary material for: A Network of Sporogenesis-Responsive Genes Regulates the Growth, Asexual Sporogenesis, Pathogenesis and Fusaric Acid Production of Fusarium oxysporum f. sp. cubense
Source: J Fungi (Basel). 2023 Dec 19;10(1):1. doi: 10.3390/jof10010001 (PMC10820103; doi:10.3390/jof10010001)
Supplement: Supplementary file 1 [file jof-10-00001-s001.zip › jof-2714693-supplementary.pdf]

Figures and tables

Supplementary Figures

Figure S1.

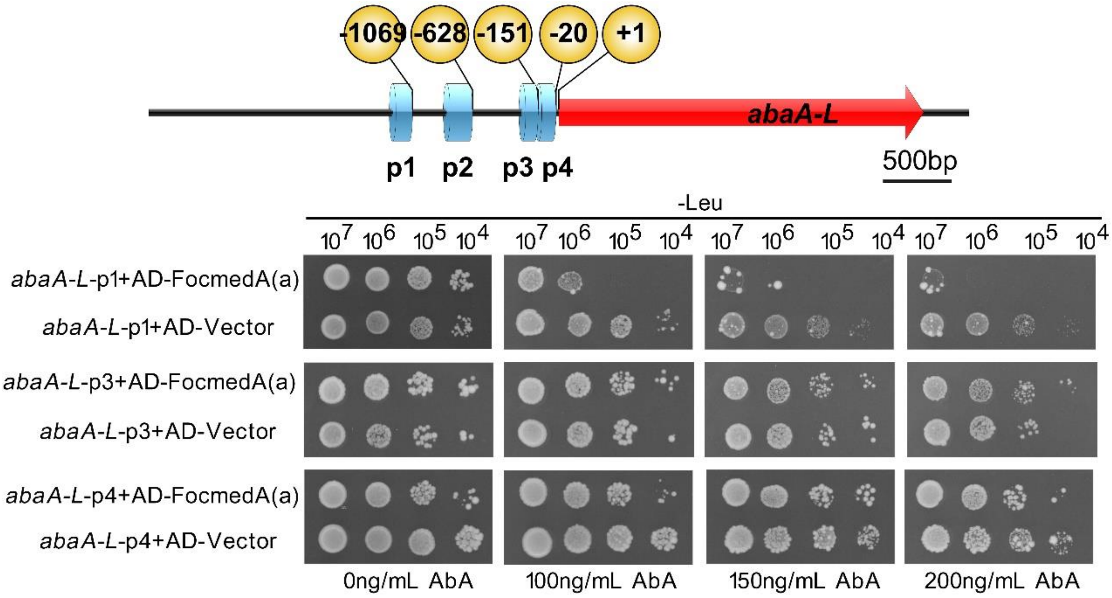

Figure S1. The Y1H technology was used to determine whether FocMedA(a) proteins interacted with *abaA-L* promoter.

Figure S2.

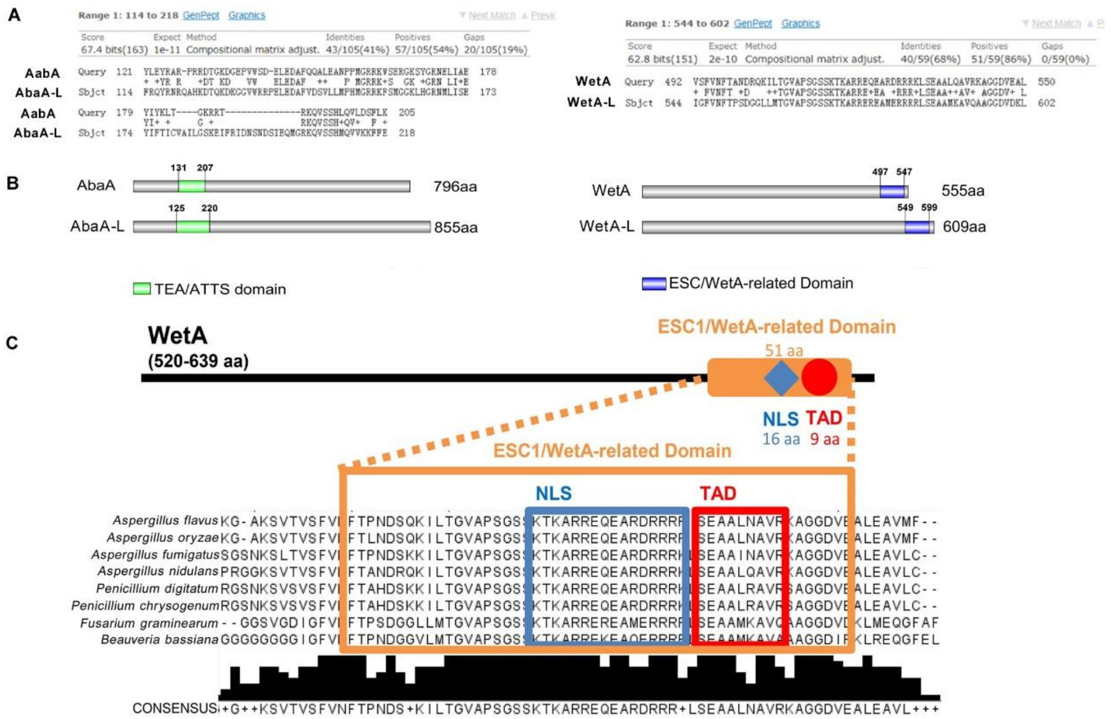

Figure S2. Similarity analysis between AbaA and AbaA-L, WetA and WetA-L. (A) Conserved residues between AbaA/WetA and their putative homologs in Foc. AbaA-L means AbaA-like protein in Foc for its low protein identity. WetA-L means WetA-like protein in Foc for its low protein identity. (B) The predicted proteins architecture and their conserved domains. (C) The consensus sequence among WetA and its homologs. This image is from the following reference: Wu MY, et al. WetA bridges cellular and chemical development in Aspergillus flavus. PLoS One. 2017, 12(6):e0179571.

Figure S3.

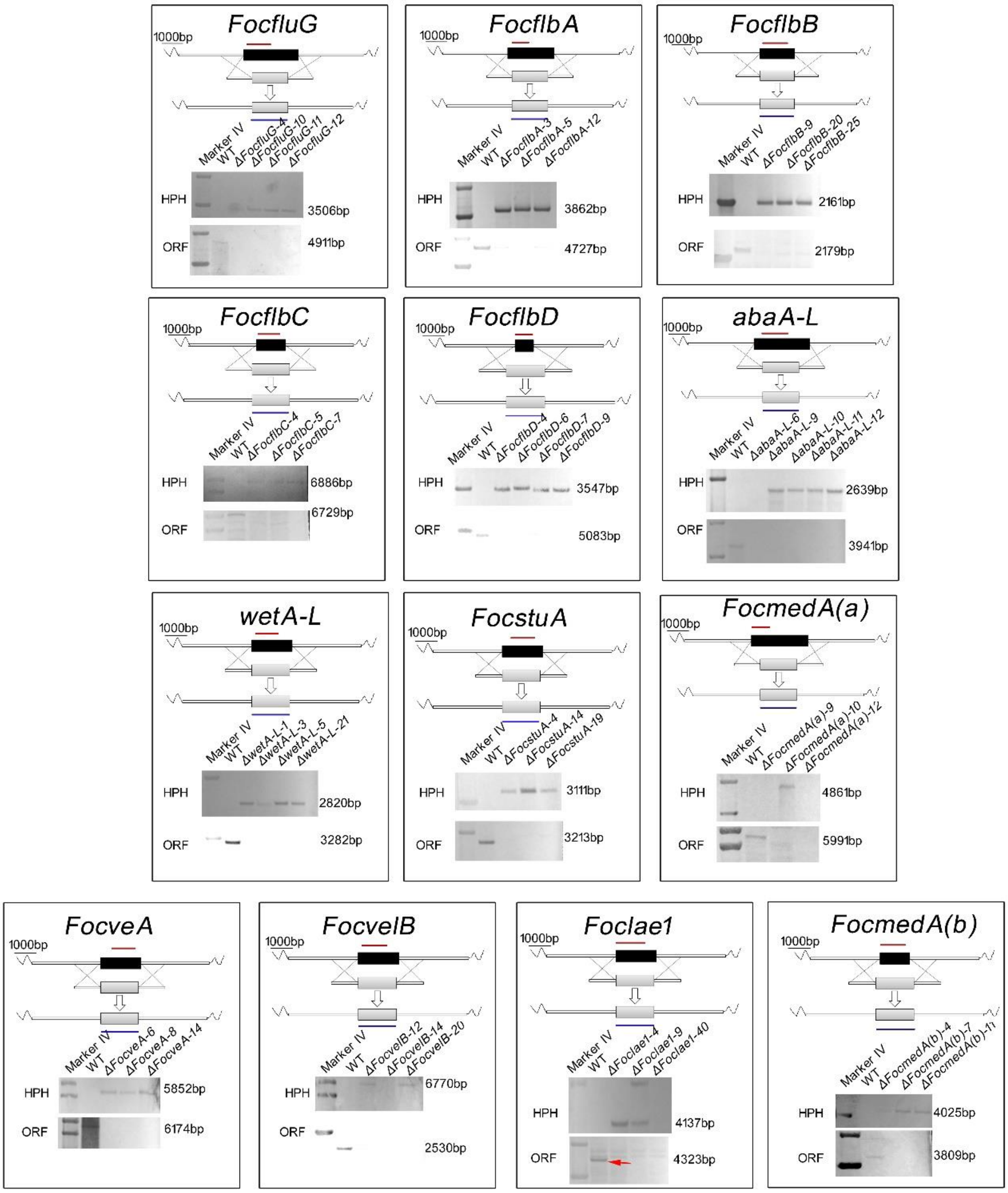

Figure S3. Southern blot hybridization analyses for gene deletion mutants of Foc obtained from this study. Top, schematic representation of the genes deletion strategy. Middle and button, the fragments of HPH (blue line)and genes ORF (red line) were used as a probe for Southern blot analyses, respectively.

Figure S4.

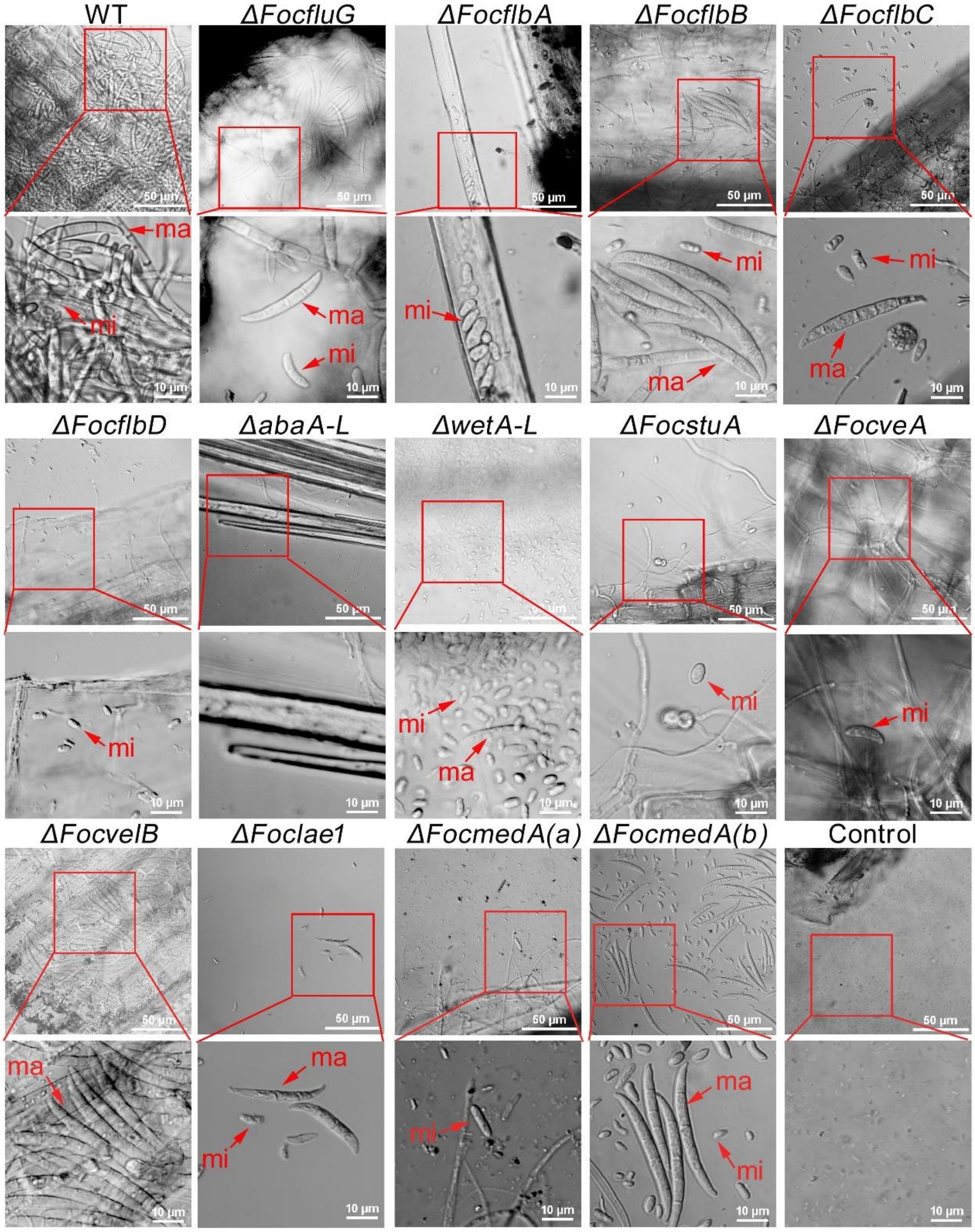

Figure S4. Microconidia and macroconidia production on banana petioles for the wild-type strain and the sporulation-related genes deletion mutants.

Figure S5.

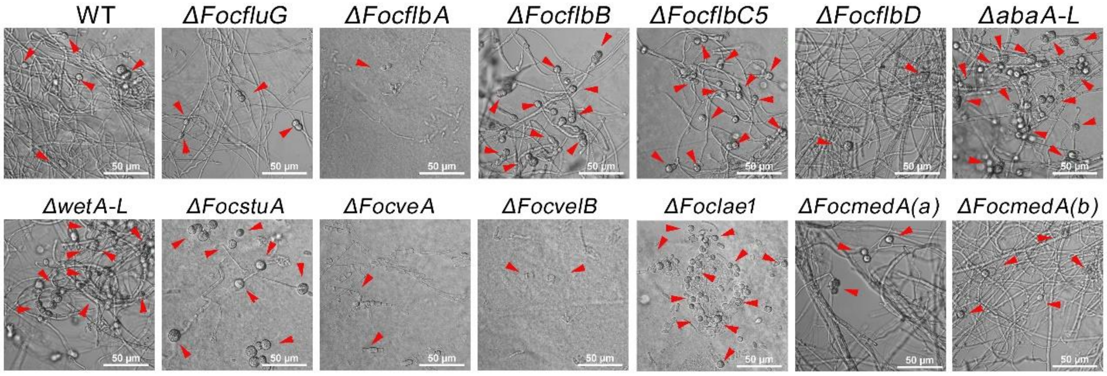

Figure S5. Chlamydospore production ability of the various mutants. Photographs were taken at the 10th day of incubation. Red arrowheads indicate chlamydospores.

Figure S6.

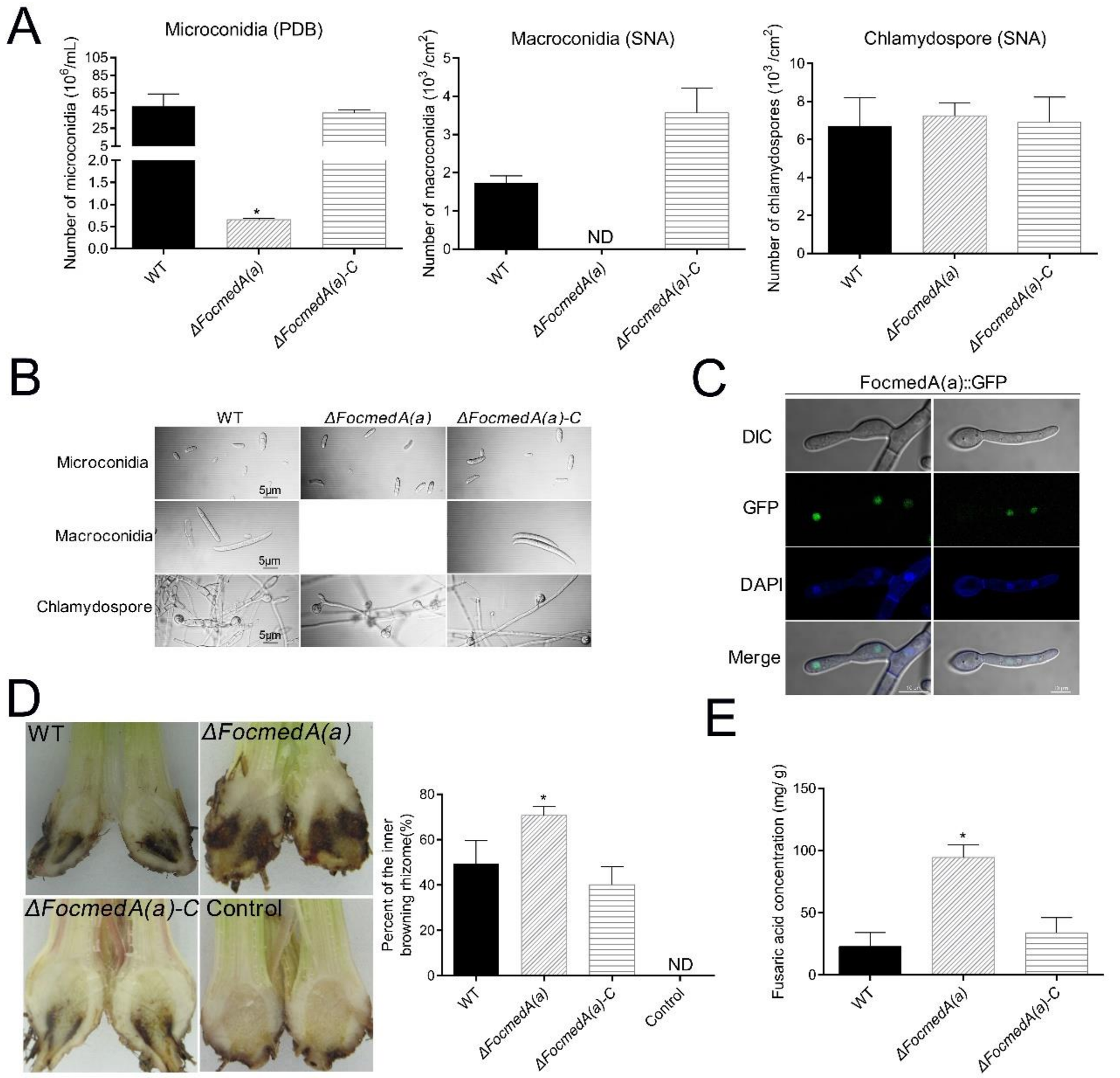

Figure S6. Roles of the *FocmedA(a)* in microconidia and macroconidia production, fusaric acid production and pathogenicity in *Fusarium oxysporum* f. sp. *cubense*. (A) Conidiation in PDB or SNA media. (B) Morphology of spores (produced in SNA) observation by microscope. (C) The subcellular localization of FocMedA(a) protein. DAPI (4',6-diamidino-2-phenylindole) is a nuclear dye. (D) Disease symptoms and percentage of inner necrosis on the infected banana corms. (E) Quantification of fusaric acid (FA) produced by the indicated strains. FA production levels are presented in mg/g dry mycelial weight. Three independent experiments were performed. Values are presented as means ± SD. Values with asterisks are significantly different according to Student's t-test at $P < 0.05$. ND means no data.

Figure S7.

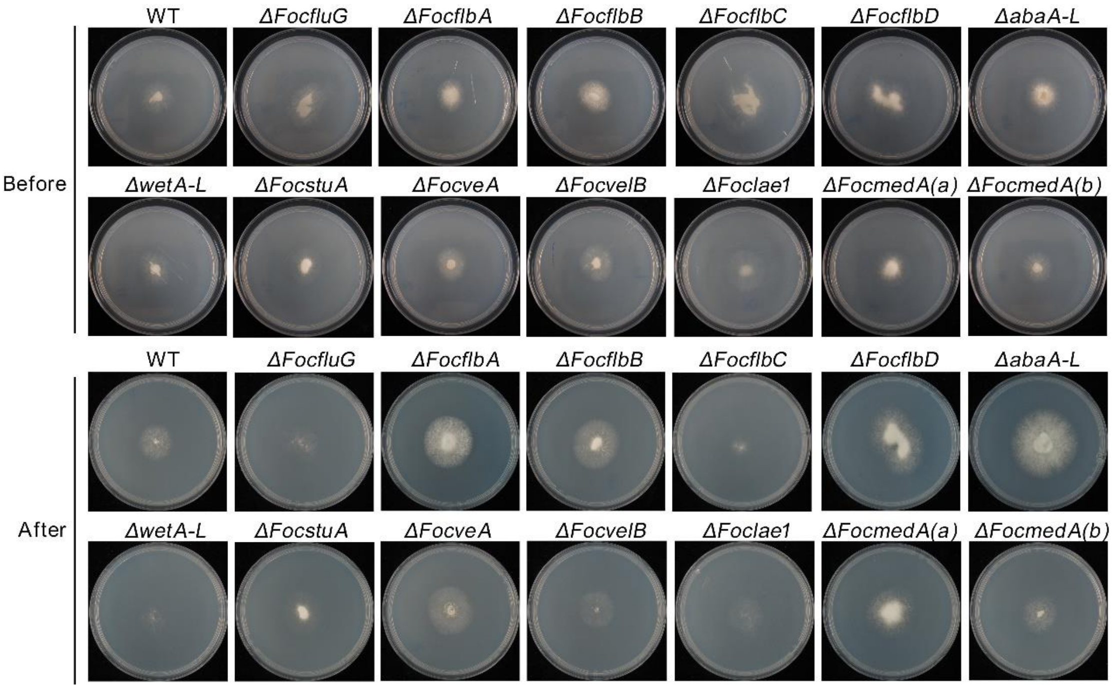

Figure S7. The conidiation-related genes are not required for cellophane membrane penetration in Foc.

Supplementary Tables

Table S1

Table S1 The primers used for genes disruption and mutants identification

Primer name	Sequence(5'-3')	Purpose
FocfluG-UP-F	TACAGTTACCCTGGAGCA	Gene deletion
FocfluG-UP-R	CATTCATTGTTGACCTCCACTAGCTCCAATGGCGTGATGACCGTTT	Gene deletion
FocfluG-DOWN-F	GCAAAGGAATAGAGTAGATGCCGACCGCATTTGATGCCCAGTGTC	Gene deletion
FocfluG-DOWN-R	CGTTGTTAGGCTCGTCTT	Gene deletion
FocfluG-NEST-F	TTGGCAGGTGAATTTGTC	Gene deletion
FocfluG-NEST-R	ACTCCTCTGATGGGCTCT	Gene deletion
FocfluG-ID-F	GCCAGCTACCCTTTCACT	Gene deletion
FocfluG-ID-R	CCTTACGAGGACCAATACG	Gene deletion
FocfluG-probe-F	TCGCCAGAAAGCAGAAGG	Southern blot probe
FocfluG-probe-R	CCAATACGAAGGCTGCTC	Southern blot probe
HPH-F	TGGAGCTAGTGGAGGTCAACA	Gene deletion/ probe
HPH-R	CGGTCGGCATCTACTCTATTC	Gene deletion/ probe
FocflbA-UP-F	GGGGTCTTTTGCTCTGGA	Gene deletion
FocflbA-UP-R	CATTCATTGTTGACCTCCACTAGCTCCAGGGGAGTTTTGCGGTGAC	Gene deletion
FocflbA-DOWN-F	GCAAAGGAATAGAGTAGATGCCGACCGGCGATGATGTGCAGGATA	Gene deletion
FocflbA-DOWN-R	CATAGATGCCGTGAGTTTT	Gene deletion
FocflbA-NEST-F	TTCCGAGACGGGCTGTTG	Gene deletion
FocflbA-NEST-R	CGGAAACTCAGTCTTCGA	Gene deletion
FoclbA-ID-F	GCCGTGTTGTCCTACCCA	Gene deletion
FocflbA-ID-R	CCGTTCCTAGCGCAGAAT	Gene deletion
FocflbA-probe-F	GCCATACGGCTTCCTCAG	Southern blot probe
FocflbA-probe-R	CAATTCCGACACTTGCTTC	Southern blot probe
FocflbB-UP-F	GATACAACCCAAATCACTTC	Gene deletion
FocflbB-UP-R	CATTCATTGTTGACCTCCACTAGCTCCAAGAAAGGACTGTCGTGGC	Gene deletion
FocflbB-DOWN-F	GCAAAGGAATAGAGTAGATGCCGACCGCCAATACATCTCCCTAAC	Gene deletion
FocflbB-DOWN-R	GCCAAGCGGTTGAAGTCG	Gene deletion
FocflbB-NEST-F	ACCCTGCCTTGTTATTGC	Gene deletion
FocflbB-NEST-R	ACCTCGGTCGTAGCCCTGTC	Gene deletion
FocflbB-ID-F	TACCGATAAGAGGACGACG	Gene deletion
FocflbB-ID-R	GGTTCGTATCGCCATCCA	Gene deletion
FocflbB-probe-F	TTACCGATAAGAGGACGACG	Southern blot probe
FocflbB-probe-R	GTGTCCTTGCCGCTGAAC	Southern blot probe
FocflbC-UP-F	CAGTGGCGTTCGTGAGTT	Gene deletion
FocflbC-UP-R	CATTCATTGTTGACCTCCACTAGCTCCAAGATGCGTGGTAGAGGGA	Gene deletion
FocflbC-DOWN-F	GCAAAGGAATAGAGTAGATGCCGACCGAGAGGCGACGCAACAACG	Gene deletion
FocflbC-DOWN-R	TCGGCTGCTATCTACTGG	Gene deletion

FocflbC-NEST-F	CCCCGTAATCCTTCTCCT	Gene deletion
FocflbC-NEST-R	TTCCCTATGTCCCTGACC	Gene deletion
FocflbC-ID-F	TGACCACGCCAGAAGATA	Gene deletion
FocflbC-ID-R	GTGAAAGGGAACATACGC	Gene deletion
FocflbC-Probe-F	CCACTCAGCAGCGGTTTG	Southern blot probe
FocflbC-Probe-R	GGTCTTCAGAGCCAGCCT	Southern blot probe
FocflbD-UP-F	GCTGGTAGCGATGGTGAC	Gene deletion
FocflbD-UP-R	CATTCATTGTTGACCTCCACTAGCTCCACAGTTTGGGCTTTGTTGC	Gene deletion
FocflbD-DOWN-F	GCAAAGGAATAGAGTAGATGCCGACCGATTCTGGTGGATGGGAGG	Gene deletion
FocflbD-DOWN-R	CAATCACAGCAACGGCAC	Gene deletion
FocflbD-NEST-F	TCCCAGTGACCGTCATAT	Gene deletion
FocflbD-NEST-R	CCTTTCGTTCGGGACACC	Gene deletion
FocflbD-ID-F	ACTCAGGCTTGCCAACAG	Gene deletion
FocflbD-ID-R	ACATATCGGGACAGACACT	Gene deletion
FocflbD-probe-F	ACAACACCGCTCCTACCC	Southern blot probe
FocflbD-probe-R	TCGGTTTTGTGATTGCTG	Southern blot probe
abaA-L-UP-F	AATGCGTTGAGGGACACC	Gene deletion
abaA-L-UP-R	CATTCATTGTTGACCTCCACTAGCTCCATCAAGACGTGGAATGGTG	Gene deletion
abaA-L-DOWN-F	GCAAAGGAATAGAGTAGATGCCGACCGTCTTTCGGAGTTGTTGGG	Gene deletion
abaA-L-DOWN-R	TATCTAACCCTCAACTCG	Gene deletion
abaA-L-NEST-F	GCAGAAGAAGGTTGGAGGAC	Gene deletion
abaA-L-NEST-R	CTCACCCATCCCTCATCC	Gene deletion
abaA-L-ID-F	CAGCAAGGGAGATGACCG	Gene deletion
abaA-L-ID-R	AAAGCTGGCCGGGTACGAC	Gene deletion
abaA-L-probe-F	TGGCTTGGACCTATACTAATG	Southern blot probe
abaA-L-probe-R	ATCGGTTGGAGTCGGGAG	Southern blot probe
wetA-L-UP-F	GGAGATCATCGCTGGACG	Gene deletion
wetA-L-UP-R	CATTCATTGTTGACCTCCACTAGCTCCATTACGGAAGACGACGAAG	Gene deletion
wetA-L-DOWN-F	GCAAAGGAATAGAGTAGATGCCGACCGGGGGAAGAATACGAGTTG	Gene deletion
wetA-L-DOWN-R	AGAAGATGTGGCAGTGGG	Gene deletion
wetA-L-NEST-F	CAACCGACCCGACTCAAA	Gene deletion
wetA-L-NEST-R	TCGCCTGAAATAGCCTCC	Gene deletion
wetA-L-ID-F	TGGACTGACGGACGAGAA	Gene deletion
wetA-L-ID-R	CGTTCTGATGTTGGAGGG	Gene deletion
wetA-L-probe-F	TCTCAGCCAGCCTCATCC	Southern blot probe
wetA-L-probe-R	CTGATGTTGGAGGGATAGGC	Southern blot probe
FocstuA-UP-F	TAACACCATGCAACTAGGCC	Gene deletion
FocstuA-UP-R	CATTCATTGTTGACCTCCACTAGCTCCAAGGAAGCAGTCGAGAAAGGA	Gene deletion
FocstuA-DOWN-F	GCAAAGGAATAGAGTAGATGCCGACCGGCGCCAAGATTCCTGTGTAA	Gene deletion
FocstuA-DOWN-R	GCTCGTGACGTTCGTGATG	Gene deletion
FocstuA-NEST-F	TGAGTCTCAGCTTGAACCCA	Gene deletion
FocstuA-NEST-R	TGGTTAGGTTGGGCACAGC	Gene deletion
FocstuA-ID-F	CCTTACGACCGTGCACTAGA	Gene deletion
FocstuA-ID-R	GTCATAAACAGGAGCAGGGG	Gene deletion
FocstuA-probe-F	GGCACTCCTTTATCATCCG	Southern blot probe
FocstuA-probe-R	GCTAATGGCAGAGGGAGG	Southern blot probe

FocveA-UP-F	TAACTATCACCCTGCCCTAT	Gene deletion
FocveA-UP-R	CATTCATTGTTGACCTCCACTAGCTCCAAAAGTCAAGTCAAGCCTATC	Gene deletion
FocveA-DOWN-F	GCAAAGGAATAGAGTAGATGCCGACCGTTGGGAATGGCAGACTTA	Gene deletion
FocveA-DOWN-R	CACTGACCTGGAATGGAA	Gene deletion
FocveA-NEST-F	ATCACGCACTGTACTCGC	Gene deletion
FocveA-NEST-R	ACTTGGCATATTTCCACC	Gene deletion
FocveA-ID-F	AAGAGGGCAAGGACATCA	Gene deletion
FocveA-ID-R	TCCATCAATCCAGGAAACT	Gene deletion
FocvelB-UP-F	GCTTGCGTTCGTTCATAT	Gene deletion
FocvelB-UP-R	CATTCATTGTTGACCTCCACTAGCTCCAAAGGTTCGGGAGGTAGGT	Gene deletion
FocvelB-DOWN-F	GCAAAGGAATAGAGTAGATGCCGACCGAGATGGGCTTTGGAAATT	Gene deletion
FocvelB-DOWN-R	TCGCTGTTCGGTCAAGTA	Gene deletion
FocvelB-NEST-F	GCCTACTTGATAAAACCCG	Gene deletion
FocvelB-NEST-R	CGCACATGGCGTAAACTC	Gene deletion
FocvelB-ID-F	AGGTCTGTTTCTTTCGTTGC	Gene deletion
FocvelB-ID-R	AGTTCAGCCCTTGCGATT	Gene deletion
FocvelB-probe-F	ACATCATAGCCAACCACCC	Southern blot probe
FocvelB-probe-R	ACGCTGAGGTCTTGGAGG	Southern blot probe
Foclae1-UP-F	GACCCATTGAATCCATCC	Gene deletion
Foclae1-UP-R	CATTCATTGTTGACCTCCACTAGCTCCA TAACGGCGATAAGTCTGG	Gene deletion
Foclae1-DOWN-F	GCAAAGGAATAGAGTAGATGCCGACCG ACAACGCAAGGAATACGA	Gene deletion
Foclae1-DOWN-R	TCAACTCCACGACATACAAG	Gene deletion
Foclae1-NEST-F	GTACCTCTAACGCTGTGC	Gene deletion
Foclae1-NEST-R	AATGGCAATATGTAGCAC	Gene deletion
Foclae1-ID-F	TAGAGTTCTTTGTGGAGCAG	Gene deletion
Foclae1-ID-R	ATTGTGGTAGACTTGAGGC	Gene deletion
Foclae1-probe-F	ACCCGCATTGCTTCTCAC	Southern blot probe
Foclae1-probe-R	ATTTGCCGATGTTCTTGC	Southern blot probe
FocmedA(a)-UP-F	ACCCGCACTGAACCATCT	Gene deletion
FocmedA(a)-UP-R	CATTCATTGTTGACCTCCACTAGCTCCAGTCCAGGGAGGCGAGTTA	Gene deletion
FocmedA(a)-DOWN-F	GCAAAGGAATAGAGTAGATGCCGACCGTCGTGTTGATGGCTTGGT	Gene deletion
FocmedA(a)-DOWN-R	GCTATTCCGTCTATCCTATGTC	Gene deletion
FocmedA(a)-NEST-F	ACAGCGATGTCCTGAAGC	Gene deletion
FocmedA(a)-NEST-R	AACGCAGTCCAATAGCAAAA	Gene deletion
FocmedA(a)-ID-F	CCTATCTCGCTTTATTCCG	Gene deletion
FocmedA(a)-ID-R	ATGCTTTGTCCAGGAGGC	Gene deletion
FocmedA(a)-probe-F	AGTTCCAGTGTCCTCCCT	Southern blot probe
FocmedA(a)-probe-R	TCAGATCCGAACACCAAA	Southern blot probe
FocmedA(b)-UP-F	TTCTTGCCTAAAGTCCTCC	Gene deletion
FocmedA(b)-UP-R	CATTCATTGTTGACCTCCACTAGCTCCAAACATGCTGTGCCTGGTC	Gene deletion
FocmedA(b)-DOWN-F	GCAAAGGAATAGAGTAGATGCCGACCGCCAAATGGCTATGGGTCG	Gene deletion
FocmedA(b)-DOWN-R	TGGCAGAGGAGCGGAAAC	Gene deletion
FocmedA(b)-ID-F	TCAGTTTCTGGAAGGGTCG	Gene deletion
FocmedA(b)-ID-R	GTTGGCTGGTCGCTCATT	Gene deletion
FocmedA(b)-NEST-F	TCCTCATCGCTGTCAACT	Gene deletion
FocmedA(b)-NEST-R	GAAGCACGGTCTTCAATT	Gene deletion

FocmedA(b)-probe-F	AAGACGGTTCTGGGTTCA	Southern blot probe
FocmedA(b)-probe-R	TCTGTGCTGCTGGGACTA	Southern blot probe

Table S2

Table S2 The primers used for qRT-PCR and vectors construction

Primer name	Sequence(5'-3')	Purpose
FocfluG-RT-F	ACGGTGAGGATGAGCACAAG	qRT-PCR
FocfluG-RT-R	AAGGGTAGCTGGCATGAAGG	qRT-PCR
FocflbA-RT-F	AATCGAGGTCATCTTCCGCC	qRT-PCR
FocflbA-RT-R	ATCTCGGTAGTCATGCAGCG	qRT-PCR
FocflbB-RT-F	ATGACAATACTGCGGCCTCC	qRT-PCR
FocflbB-RT-R	GTGTTCTCTGTGCCTGACGA	qRT-PCR
FocflbC-RT-F	CTGATGACCACGCCAGAAGA	qRT-PCR
FocflbC-RT-R	GCGCCATAGATAGGTCGAGG	qRT-PCR
FocflbD-RT-F	TCCGTGGTCACAGTTTGAGG	qRT-PCR
FocflbD-RT-R	TCAGGTGTGATGGGTTCGTG	qRT-PCR
abaA-L-RT-F	CGCAGTCACACAGCTTCAAC	qRT-PCR
abaA-L-RT-R	GGTTGGTTCCTGGGTGTAGG	qRT-PCR
wetA-L-RT-F	TCCAATGGGTCTGTCGCATC	qRT-PCR
wetA-L-RT-R	CCTGTCATTAGGAGACCGCC	qRT-PCR
Focstua-RT-F	AGCGACACAAGACCATGGAG	qRT-PCR
Focstua-RT-R	TACACCGGGGCAGAAGGATA	qRT-PCR
Focvea-probe-F	ACCTTTCTGTCCGCCACG	qRT-PCR
Focvea-probe-R	GGTTGAACTGGACGGAGC	qRT-PCR
FocvelB-RT-F	TGTTGTCGGTGGATCTGTGG	qRT-PCR
FocvelB-RT-R	TTGCCCATATCCAGACTCGC	qRT-PCR
Foclae1-RT-F	CTGGATACCCCGATGTGACG	qRT-PCR
Foclae1-RT-R	GACTCTGACACTGCGGTTGA	qRT-PCR
FocmedA(a)-RT-F	CGATCAGCCGAGTACCTCAC	qRT-PCR
FocmedA(a)-RT-R	GTAGTGAGGGTCCGTGAAGC	qRT-PCR
FocmedA(b)-RT-F	TATCCAAGGCCAAACCCGAC	qRT-PCR
FocmedA(b)-RT-R	CTTCTGTGCTGCTGGGACTA	qRT-PCR
FocActin-RT-F	CCCATCAACCCCAAGTCCAA	qRT-PCR
FocActin-RT-R	GAAGGGCGAAACCCTCGTAA	qRT-PCR
pAbAi-abaA-L-p1-F	cttgaattcgagctcggtaccAATGCGTTGAGGGACACC	Y1H

pAbAi-abaA-L-p1-R	agcacatgcctcgaggtcgac ATTGACCCTACTCCTCCC	Y1H
pAbAi-abaA-L-p2-F	cttgaattcgagctcggtaccAACGCTTATTCGGAGTGG	Y1H
pAbAi-abaA-L-p2-R	agcacatgcctcgaggtcgacTGCTAACCCAACCTGACC	Y1H
pAbAi-abaA-L-p3-F	cttgaattcgagctcggtaccCTGGTCCGCAGACTATCA	Y1H
pAbAi-abaA-L-p3-R	agcacatgcctcgaggtcgac AAGACGTGGAATGGTGGG	Y1H
pAbAi-abaA-L-p4-F	cttgaattcgagctcggtaccCTATCCCACCATTCCACG	Y1H
pAbAi-abaA-L-p4-R	agcacatgcctcgaggtcgacGCAAAGGAATGCCGACTG	Y1H
pAbAi-F	AAGCTTGAATTCGAGCTCG	Y1H
pAbAi-R	ATCCCAAGAAGATGTAATGC	Y1H
pGADT7-FocmedA(a)-F	gtaccagattacgctcatatgATGTCTACTGTCAAGTTCCAGTGTCC	Y1H
pGADT7-FocmedA(a)-R	atgcccacccgggtggaattcTCATGCTCTTGGTGCAGGTTC	Y1H
T7 (F)	TAATACGACTCACTATAGGGC	Y1H
3AD (R)	AGATGGTGCACGATGCACAG	Y1H
abaA-L-CRT-P1-F	GAAGCCGAGAACGCACTAAA	Chip-qPCR
abaA-L-CRT-P1-R	AACCTTGTTCACCTGTCCTCC	Chip-qPCR
abaA-L-CRT-P2-F	CCAAACCTTCTGTACCTTGCG	Chip-qPCR
abaA-L-CRT-P2-R	CATGAACCAAGGGACTGTGGA	Chip-qPCR
abaA-L-CRT-CK-F	CTCTTATCTCAGTCCGCCCC	Chip-qPCR
abaA-L-CRT-CK-R	CTTGTTCAGGTGGGGGAAGG	Chip-qPCR

Table S3

Table S3 The strains used in this study

**Strain**	**Abbreviated name**	**Genotype**	**Source**
WT-58	WT	Foc TR4	This study
ΔFocfluG-12	*ΔFocfluG*	*FocfluG* deletion mutant of Foc TR4	This study
ΔFocflbA-12	*ΔFocflbA*	*FocflbA* deletion mutant of Foc TR4	This study
ΔFocflbB-20	*ΔFocflbB*	*FocflbB* deletion mutant of Foc TR4	This study
ΔFocflbC-5	*ΔFocflbC*	*FocflbC* deletion mutant of Foc TR4	This study
ΔFocflbD-6	*ΔFocflbD*	*FocflbD* deletion mutant of Foc TR4	This study
ΔabaA-L-10	*ΔabaA-L*	*abaA-L* deletion mutant of Foc TR4	This study
ΔwetA-L-1	*ΔwetA-L*	*wetA-L* deletion mutant of Foc TR4	This study
ΔFocstuA-4	*ΔFocstuA*	*FocstuA* deletion mutant of Foc TR4	This study
ΔFocveA-6	*ΔFocveA*	*FocveA* deletion mutant of Foc TR4	This study
ΔFocvelB-12	*ΔFocvelB*	*FocvelB* deletion mutant of Foc TR4	This study
ΔFoclae1-4	*ΔFoclae1*	*Foclae1* deletion mutant of Foc TR4	This study
ΔFocmedA(a)-10	*ΔFocmedA(a)*	*FocmedA(a)* deletion mutant of Foc TR4	This study
ΔFocmedA(b)-11	*ΔFocmedA(b)*	*FocmedA(b)* deletion mutant of Foc TR4	This study
ΔFocmedA(a)-C-GFP	*ΔFocmedA(a)-C*	Complementation, usded for ChIP-qPCR assay	This study
